# Comparative and functional genomics reveals genetic diversity and determinants of host specificity among reference strains and a large collection of Chinese isolates of the phytopathogen *Xanthomonas campestris *pv. *campestris*

**DOI:** 10.1186/gb-2007-8-10-r218

**Published:** 2007-10-10

**Authors:** Yong-Qiang He, Liang Zhang, Bo-Le Jiang, Zheng-Chun Zhang, Rong-Qi Xu, Dong-Jie Tang, Jing Qin, Wei Jiang, Xia Zhang, Jie Liao, Jin-Ru Cao, Sui-Sheng Zhang, Mei-Liang Wei, Xiao-Xia Liang, Guang-Tao Lu, Jia-Xun Feng, Baoshan Chen, Jing Cheng, Ji-Liang Tang

**Affiliations:** 1 Guangxi Key Laboratory of Subtropical Bioresources Conservation and Utilization, and College of Life Science and Technology, Guangxi University, Daxue Road, Nanning, Guangxi 530004, People's Republic of China; 2 CapitalBio Corporation, Life Science Parkway, Changping District, Beijing 102206, People's Republic of China

## Abstract

**Background:**

*Xanthomonas campestris *pathovar *campestris *(*Xcc*) is the causal agent of black rot disease of crucifers worldwide. The molecular genetic diversity and host specificity of *Xcc *are poorly understood.

**Results:**

We constructed a microarray based on the complete genome sequence of *Xcc *strain 8004 and investigated the genetic diversity and host specificity of *Xcc *by array-based comparative genome hybridization analyses of 18 virulent strains. The results demonstrate that a genetic core comprising 3,405 of the 4,186 coding sequences (CDSs) spotted on the array are conserved and a flexible gene pool with 730 CDSs is absent/highly divergent (AHD). The results also revealed that 258 of the 304 proved/presumed pathogenicity genes are conserved and 46 are AHD. The conserved pathogenicity genes include mainly the genes involved in type I, II and III secretion systems, the quorum sensing system, extracellular enzymes and polysaccharide production, as well as many other proved pathogenicity genes, while the AHD CDSs contain the genes encoding type IV secretion system (T4SS) and type III-effectors. A *Xcc *T4SS-deletion mutant displayed the same virulence as wild type. Furthermore, three avirulence genes (*avrXccC*, *avrXccE1 *and *avrBs1*) were identified. *avrXccC *and *avrXccE1 *conferred avirulence on the hosts mustard cultivar Guangtou and Chinese cabbage cultivar Zhongbai-83, respectively, and *avrBs1 *conferred hypersensitive response on the nonhost pepper ECW10R.

**Conclusion:**

About 80% of the *Xcc *CDSs, including 258 proved/presumed pathogenicity genes, is conserved in different strains. *Xcc *T4SS is not involved in pathogenicity. An efficient strategy to identify *avr *genes determining host specificity from the AHD genes was developed.

## Abstract 2

Construction of a microarray based on the genome of Xanthomonas campestris pv.campestris (Xcc), and its use to analyse 18 other virulent Xcc strains, revealed insights into the genetic diversity and determinants of host specificity of Xcc strains.

## Background

*Xanthomonas campestris *pathovar *campestris *(*Xcc*) is the causal agent of black rot disease, one of the most destructive diseases of cruciferous plants worldwide [1]. This pathogen infects almost all the members of the crucifer family (Brassicaceae), including important vegetables such as broccoli, cabbage, cauliflower, mustard, radish, and the major oil crop rape, as well as the model plant *Arabidopsis thaliana*. Since the late 1980s, black rot disease has become more prevalent and caused severe losses in vegetable and edible oil production in China [2,3], Nepal [4], Russia [5], Tanzania [6], and the United Kingdom [7].

It has been shown that *Xcc *is composed of genetically, serologically and pathogenically diverse groups of strains [4,8,9]. Certain *Xcc *strains are able to cause disease only in certain host plants, indicating that there are incompatible interactions between *Xcc *strains and their host plants. Flor's gene-for-gene theory [10] suggested that such an incompatible interaction between microbial pathogens and plants determines the pathogens' host specificity and is governed by an avirulence (*avr*) gene of a pathogen and the cognate resistance (*R*) gene of a host. Since the early 1980s, *Xcc *has been used as a model organism for studying plant-pathogen interactions [11-14] and more than one hundred *Xcc *pathogenicity-related genes have been identified [13,15-19]. However, few *avr *genes have been functionally characterized from *Xcc*. Recently, whole genome sequences of two *Xcc *strains, ATCC33913 [20] and 8004 [21], have been determined. Genome annotation predicted that *Xcc *possesses at least eight genes that show sequence homology to the known *avr *genes discovered from other bacteria [20,21]. Mutagenesis analysis of these eight *avr*-homologous genes detected avirulence activity for only *avrXccFM *[22].

Comparison of the whole genome sequences of the strains 8004 and ATCC33913 has revealed that the two genomes are highly conserved with respect to gene content [20,21]. There are only 72,521 bp and 5 protein-coding sequences (CDSs) different between their genomic sizes and their total predicted CDSs, respectively [20,21]. Although 170 strain-specific CDSs (108 specific for strain 8004 and 62 for strain ATCC33913) were identified and three of the 8004 strain-specific CDSs were found to be involved in virulence [20,21], the genetic basis about the host specificity of *Xcc *remains unclear. As both strains 8004 and ATCC33913 were isolated from the UK [20,21], they might be closely related strains sharing a late common ancestor and this small genetic variability might not represent the nature of *Xcc *genetic diversity. To further determine the genetic variability and host specificity of *Xcc*, in this work we collected 18 *Xcc *virulent strains isolated from different host plants and different geographical areas from North China to South China and compared their genomes with the sequences of strain 8004 by array-based comparative genome hybridization (aCGH).

The aCGH analysis has been used to study bacterial pathogenicity, genetic diversity and evolution [23-31]. This approach facilitates the comparison of un-sequenced bacterial genomes with a sequenced reference genome of a related strain or species. Genes in the organisms under study are categorized into 'present' and 'absent/divergent' categories based on the level of hybridization signal. The resolution threshold of the aCGH is generally at the single gene level (gene-specific microarray) [32], which is just appropriate for identifying the genetic determinants responsible for host specificity of plant pathogens that follow the gene-for-gene relationship. This genomotyping technique has been used to analyze phytopathogenic bacterial strain variation in *Xylella fastidiosa *[33,34] and *Ralstonia solanacearum *[35].

In this paper we report the identification of a common genome backbone and a flexible gene pool of *Xcc *revealed by aCGH analysis. We also demonstrate that the type IV secretion system (T4SS), which has been shown or proposed to be involved in virulence of several bacterial pathogens [36-40], is not engaged in the virulence of *Xcc*. Furthermore, three *avr *genes were identified from the flexible gene pool by analysis of the correlations between the occurrence of genes and the reaction of different strains in different hosts followed by experimental functional confirmation.

## Results

### Characterization of Chinese isolates as *Xcc*

Twenty-two different strains/isolates were collected for this study. Of these, the *Xcc *strain ATCC33913 is a type strain, isolated from Brussels sprout (*Brassica oleracea *var. *gemmifera*) in the UK in 1957 [20], and the *Xcc *strain 8004 is a laboratory strain with spontaneous rifampicin-resistance, derived from *Xcc *NCPPB No.1145 isolated from cauliflower (*B. oleracea *var. *botrytis*) in the UK in 1958 [14]. The other 20 isolates were collected from different infected cruciferous plants in various geographic locations over a wide range of latitudes across China and named CN01 to CN20 (Table 1). These isolates were validated by morphological, virulent and molecular analyses. All the isolates formed typical *X. campestris *colonies of yellow mucoid texture on NYG agar medium [14] and caused typical black rot disease symptoms on the host plant radish (*Raphanus sativus *var. *radicula*; data not shown). To further confirm the isolates, their partial 16S-23S rDNA intergenic spacer (ITS) regions [41] were examined by PCR and sequencing. A PCR fragment 464 bp in length was obtained for every isolate except CN13 and CN19, for which no PCR product was obtained. Sequencing results showed that five isolates have identical ITS sequences to that of strain 8004, while the ITS sequences of the other 13 isolates differ from that of 8004 by only one or two nucleotides (Additional data files 1 and 2). The isolates CN13 and CN19 were not used for further study in this work as they were not confirmed to be *Xcc *by the 16S-23S rDNA ITS analysis. The phylogenetic analysis by the maximal parsimony method [42] showed that the 18 proven *Xcc *isolates were grouped into two clusters and each cluster contains previously identified *Xcc *strains (Additional data file 2). These two groups were significantly distinguished from other *Xanthomonas *species and *X. campestris *pathovars (Additional data file 2), further confirming the 18 isolates as *Xcc *at the molecular level. The word 'strain' will be used for the identified *Xcc *'isolates' hereafter.

**Table 1:** The origin of the *Xcc *strains used in this study

		Geographical origin	
		
Strains	Host of origin	Location (time)	Geographical coordinates*
Lab strain: 8004	Cauliflower (*Brassica oleracea *var. *botrytis*)	Sussex, UK (1958)	(0E,51.0000N)
Type strain: ATCC33913	Brussels sprout (*B. oleracea *var. *gemmifera*)	UK (1957)	(0E,52.0000N)
Chinese strains			
CN01	Chinese cabbage (*B. rapa *subsp. *pekinensis*)	Haerbin, China (2002)	126.5192E,45.6534N
CN02	Chinese cabbage (*B. rapa *subsp. *pekinensis*)	Changchun, China (2002)	125.4247E,43.7408N
CN03	Chinese cabbage (*B. rapa *subsp. *pekinensis*)	Dalian, China (2002)	121.4837E,38.9351N
CN04	Oilseed rape (*B. napus *ssp. *oleifera*)	Huhehaote, China (2002)	111.7378E,40.8792N
CN05	Chinese cabbage (*B. rapa *subsp. *pekinensis*)	Daxing, China (2002)	116.3345E,39.7243N
CN06	Chinese cabbage (*B. rapa *subsp. *pekinensis*)	Shunyi, China (2002)	116.6559E,40.1351N
CN07	Chinese cabbage (*B. rapa *subsp. *pekinensis*)	Tianjin, China (2002)	112.6522E,37.8955N
CN08	Radish (*Raphanus sativus *var. *longipinnatus*)	Taiyuan, China (2002)	117.0037E,39.2864N
CN09	Chinese cabbage (*B. rapa *subsp. *pekinensis*)	Xi'an, China (2002)	108.9551E,34.5450N
CN10	Chinese cabbage (*B. rapa *subsp. *pekinensis*)	Duqu, China (2002)	108.1164E,33.9359N
CN11	Cabbage (*B. oleracea *var. *capitata*)	Nanyang, China (2002)	112.9521E,33.0564N
CN12	Oilseed rape (*B. napus *subsp. *oleifera*)	Wuhan, China (2002)	114.4438E,30.4801N
CN14	Leaf mustard (*B. juncea *var. *foliosa*)	Guilin, China (2003)	110.3181E,25.2582N
CN15	Chinese cabbage (*B. rapa *subsp. *chinensis*)	Guilin, China (2003)	110.3207E,25.3817N
CN16	Chinese cabbage (*B. rapa *subsp. *pekinensis*)	Guilin, China (2003)	110.0797E,25.2467N
CN17	Chinese cabbage (*B. rapa *subsp. *chinensis*)	Nanning, China (2003)	108.3876E,22.8374N
CN18	Leaf mustard (*B. juncea *var. *foliosa*)	Nanning, China (2003)	108.2181E,22.8018N
CN20	Chinese kale (*B. oleracea *var. *alboglabra*)	Nanning, China (2003)	108.2865E,22.8874N

*The geographic coordinates of the *Xcc *strains in parentheses are estimated from information originating in the National Collection of Plant Pathogenic Bacteria.

### The virulence and hypersensitive response of *Xcc *strains on different plants

The *in planta *pathogenicity test of *Xcc *strains was carried out by the leaf-clipping inoculation method on eleven different cultivars (cv.) of four cruciferous species (see Materials and methods). The results showed that seven of the eleven cultivars were susceptible to all of the *Xcc *strains tested, whereas the other four plants manifested resistance to particular *Xcc *strains (Tables 1 and 2). Based upon these results, a gene-for-gene relationship governing the outcome of the interactions between the *Xcc *strains and the host plants could be postulated (Table 3). The key essentials are: first, the host plants that were susceptible to all of the *Xcc *strains possess no resistance genes against the *Xcc *strains; second, mustard cv. Guangtou possesses a resistance (*R*) gene, arbitrarily designated *Rc1*, for which the postulated interacting avirulence (*avr*) gene is designated *avrRc1*, present in strains 8004, ATCC33913, CN03, CN07, CN09, CN10, CN11, and CN20; third, cabbage cv. Jingfeng-1 and radish cv. Huaye possess an *R *gene named *Rc2 *that interacts with an *avr *gene named *avrRc2*, present in strains ATCC33913, CN03, CN14, CN15, and CN16; and fourth, Chinese cabbage cv. Zhongbai-83 possesses an *R *gene, *Rc3*, that interacts with the postulated *avrRc3 *in strains 8004, ATCC33913, CN02, CN03, CN06, CN07, CN08, CN12, CN14, CN15, CN16, CN18, as well as CN20 (Tables 2 and 3).

**Table 2:** The plant assay results of *Xcc *strains

	Plant assays*											
	
*Xcc *strains	TP1	TP2	TP3	TP4	TP5	TP6	TP7	TP8	TP9	TP10	TP11	TP12
Lab strain: 8004	-	+	+	+	+	+	+	-	+	+	+	HR
Type strain: ATCC33913	-	+	-	+	+	+	+	-	-	+	+	HR
Chinese strains												
CN01	(+)	+	+	+	+	+	+	+	+	+	+	HR
CN02	+	+	+	+	+	+	+	-	+	+	+	N
CN03	-	(+)	-	(+)	(+)	(+)	(+)	-	-	(+)	(+)	HR
CN04	+	+	+	+	+	+	+	(+)	+	+	+	N
CN05	+	+	+	+	+	+	+	+	+	+	+	N
CN06	+	+	+	+	+	+	+	-	+	+	+	N
CN07	-	+	+	+	+	+	+	-	+	+	+	N
CN08	+	+	+	+	+	+	+	-	+	+	+	N
CN09	-	+	+	+	+	+	(+)	+	+	+	+	HR
CN10	-	+	+	+	+	+	(+)	+	+	+	+	HR
CN11	-	+	+	+	+	+	+	+	+	+	+	HR
CN12	+	+	(+)	+	+	+	+	-	-	+	+	N
CN14	+	+	-	+	+	+	(+)	-	-	+	+	N
CN15	+	+	-	+	+	+	+	-	-	+	+	N
CN16	+	+	-	+	+	+	+	-	-	+	+	N
CN17	+	+	+	+	+	+	+	(+)	+	+	+	N
CN18	+	+	-	+	+	+	+	-	+	+	+	N
CN20	-	+	+	+	+	+	+	-	+	+	+	HR

*The plants used for pathogenicity test. TP1, mustard (*B. juncea *var. *megarrhiza *Tsen et Lee) cv. Guangtou; TP2, Chinese kale (*B. oleracea *var. *alboglabra*) cv. Xianggangbaihua; TP3, cabbage (*B. oleracea *var. *capitata*) cultivar (cv.) Jingfeng-1; TP4, kohlrabi (*B. oleracea *var. *gongylodes*) cv. Chunqiu; TP5, pakchoi cabbage (*B. rapa *subsp. *chinensis*) cv. Jinchengteai; TP6, pakchoi cabbage (*B. rapa *subsp. *chinensis*) cv. Naibaicai; TP7, Chinese cabbage (*B. rapa *subsp. *pekinensis*) cv. Zhongbai-4; TP8, Chinese cabbage (*B. rapa *subsp. *pekinensis*) cv. Zhongbai-83; TP9, radish (*R. sativus *var. *longipinnatus*) cv. Huaye; TP10, radish (*R. sativus *var. *radicula*) cv. Manshenghong; TP11, radish (*R. sativus *var. *sativus*) cv. Cherry Belle. +, virulent; -, non-pathogenic; (+), weakly virulent. The hypersensitive reaction (HR) tests of *Xcc *strains were carried out on non-host plant pepper (*Capsicum annuum *v. *latum*) ECW10R (TP12). HR, positive HR result; N, no HR.

**Table 3:** Postulated gene-for-gene model to explain the relationship between *Xcc *strains and the plants used*

	Resistant genes				Postulated avirulence genes in *Xcc *strains tested			
		
Plants^†^	*Rc1*	*Rc2*	*Rc3*	*Rp1*	*avrRc1*	*avrRc2*	*avrRc3*	*avrRp1*
TP1	*Rc1*	...	...	...	-	+	+	...
TP2	...	...	...	...	+	+	+	...
TP3	...	*Rc2*	...	...	+	-	+	...
TP4	...	...	...	...	+	+	+	...
TP5	...	...	...	...	+	+	+	...
TP6	...	...	...	...	+	+	+	...
TP7	...	...	...	...	+	+	+	...
TP8	...	...	*Rc3*	...	+	+	-	...
TP9	...	*Rc2*	...	...	+	-	+	...
TP10	...	...	...	...	+	+	+	...
TP11	...	...	...	...	+	+	+	...
TP12	...	...	...	*Rp1*	...	...		HR

*+, compatible interaction (susceptibility); -, incompatible interaction (resistance); ..., data unavailable. ^†^The plants used for pathogenicity test. TP1, mustard (*B. juncea *var. *megarrhiza *Tsen et Lee) cv. Guangtou; TP2, Chinese kale (*B. oleracea *var. *alboglabra*) cv. Xianggangbaihua; TP3, cabbage (*B. oleracea *var. *capitata*) cultivar (cv.) Jingfeng-1; TP4, kohlrabi (*B. oleracea *var. *gongylodes*) cv. Chunqiu; TP5, pakchoi cabbage (*B. rapa *subsp. *chinensis*) cv. Jinchengteai; TP6, pakchoi cabbage (*B. rapa *subsp. *chinensis*) cv. Naibaicai; TP7, Chinese cabbage (*B. rapa *subsp. *pekinensis*) cv. Zhongbai-4; TP8, Chinese cabbage (*B. rapa *subsp. *pekinensis*) cv. Zhongbai-83; TP9, radish (*R. sativus *var. *longipinnatus*) cv. Huaye; TP10, radish (*R. sativus *var. *radicula*) cv. Manshenghong; TP11, radish (*R. sativus *var. *sativus*) cv. Cherry Belle; TP12, non-host plant pepper (*Capsicum annuum *v. *latum*) ECW10R.

We also examined the hypersensitive response (HR) [43] of the *Xcc *strains on the nonhost pepper ECW10R, a plant commonly used to test the HR of *Xcc*. The results showed that eight hours after inoculation strains 8004, ATCC33913, CN01, CN03, CN09, CN10, CN11, and CN20 elicited a typical HR while the others did not (Table 2). According to the results, we postulated that strains 8004, ATCC33913, CN01, CN03, CN09, CN10, CN11, and CN20 possess an avirulence gene, designated *avrRp1*, that interacts with a cognate resistance gene, named *Rp1*, in the non-host plant pepper ECW10R (Tables 2 and 3).

### Sensitivity of aCGH analysis

To investigate genetic similarity and diversity among *Xcc *strains, a DNA microarray encompassing 4,186 CDSs was constructed, representing all CDSs (non-redundant) in the reference strain 8004 [21]. Primer design was based on the genomic sequence of 8004, which is composed of 4,273 CDSs [21]. Of the 4,186 CDSs, gel electrophoresis revealed successful amplification of 4,043 CDSs, representing 96.58% of the non-redundant genome content. For the CDSs predicted to be less than 100 bp in length, for which optimized primers could not be designed, and those for which PCR amplification did not work, a 70-mer oligo probe for each CDS was designed. The word 'gene' will be used in reference to the CDS that each spot corresponds to unless otherwise indicated.

**Figure 1:**
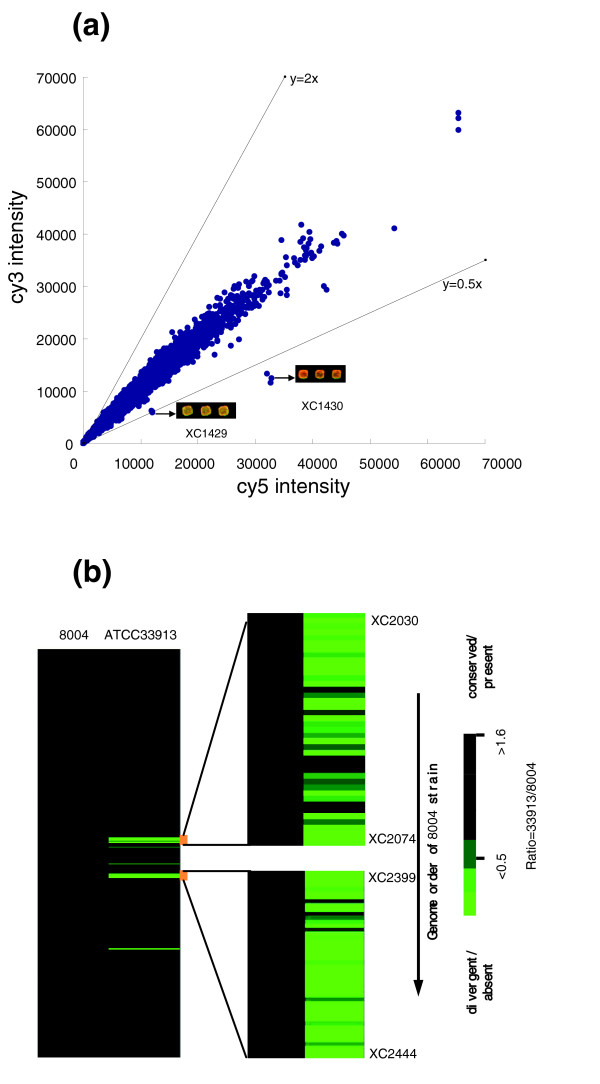
Sensitivity determination of aCGH analyses. **(a) **aCGH analyses of the reference strain 8004 and its derivative strain C1430nk. The strain C1430 possesses one extra DNA copy of the ORFs *XC1429 *and *XC1430 *compared to the reference strain 8004. **(b) **TreeView display of the aCGH clustering result of the two sequenced genomes of the *Xcc *strains 8004 and ATCC33913. Each row corresponds to the specific ORFs on the array and the ORFs are arranged in the genome order of the reference strain 8004 from *XC0001 *at the top to *XC4332 *at the bottom. From the aCGH result, it is observed that the ATCC33913 is missing two prominent DNA fragments, one from strain 8004 ORF *XC2030 *to *XC2074 *and the other from *XC2399 *to *XC2444*, which is consistent with sequence information.

To determine the sensitivity of our aCGH analysis, self-to-self hybridization was performed using genomic DNA of the reference strain 8004. After removal of faint spots for which the intensity was lower than the average plus two standard deviations of the negative controls (blank spotting solution) on the array, it was found that more than 95% of all genes on the array could be detected and the intensity ratio of the detected genes lay between 0.6 and 1.6. aCGH analyses were then carried out using the reference strain 8004 and its derivative strain C1430nk, described previously [44]. The strain C1430nk is derived from 8004 and harbors the cosmid pLAFR6 containing the open reading frames (ORFs) *XC1429 *and *XC1430*. The aCGH results revealed that only two genes, *XC1429 *and *XC1430*, had an intensity ratio of approximately 1.9-2.4 (C1430nk/8004), indicating that sole copy alteration at the genomic scale could be detected in this study (Figure 1). Based on the above results, it was presumed that the microarray can detect the 1.6-fold alteration when ignoring sequence diversity. After passing the initial tests, aCGH analyses were performed using the fully sequenced *Xcc *strains 8004 and ATCC33913. The results showed a good agreement with the complete genome sequences of 8004 and ATCC33913 (Figure 1). It was found that for the genes of strain ATCC33913, whose sequences are >90% identical to those of strain 8004, 99% of their spots on the array showed intensity ratios ≥0.5. Therefore, intensity ratios ≥0.5 were selected to be the threshold for genes detected as present/conserved within strain 8004. Furthermore, 98% of the genes previously reported to be specific to strain 8004 (that is, that are absent in the genome of strain ATCC33913) were detected as absent genes in the aCGH analysis of strain ATCC33913 (Figure 1). Our selected threshold for conserved genes here is similar to that described by Taboada *et al*. [30], who used a Log_2 _ratio (sample/reference) threshold of -0.8 to detect conserved genes in aCGH analyses with an acceptable level of false positives.

The validity of the aCGH results was further tested by PCR examination of the presence or absence of 30 genes showing a range of ratios in the aCGH analysis. The PCR primers used and PCR results are presented in Additional data file 3. The results show that a ratio (sample/8004 strain) of <0.5 gives high confidence (98%) that the gene is absent/highly divergent (AHD) in the sample strain.

### Overview of the aCGH analyses of different *Xcc *strains

**Figure 2:**
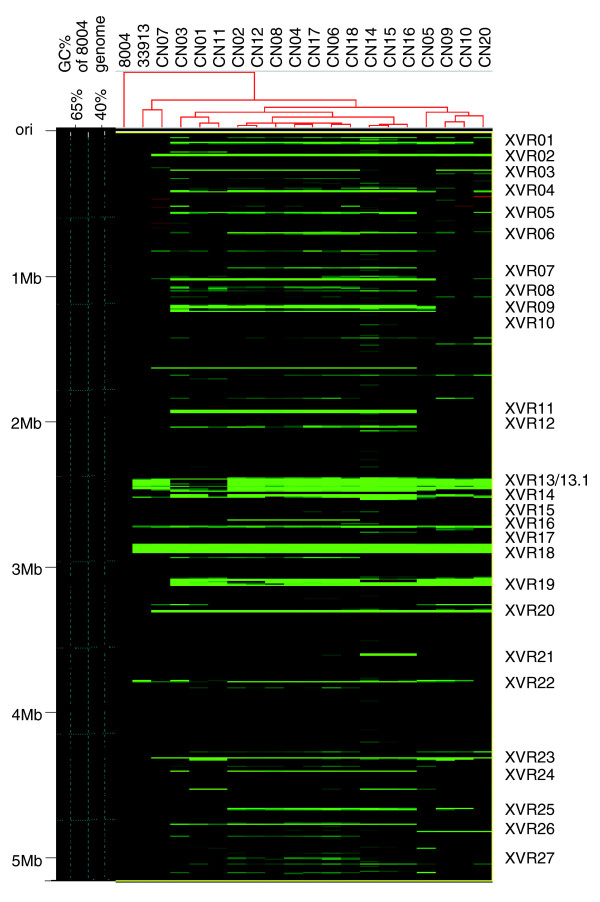
Schematic representation of the genome composition of *Xcc *strains based on aCGH analyses. The left-most line indicates the physical map scaled in megabases from the first base, the start of the putative replication origin. The curve indicates the GC content in the genome of strain 8004. The image of the hierarchical clustering was based on the aCGH results of 20 *Xcc *strains. The number of *Xcc *strains on the top shows that each column indicates each strain. Each tiny line indicates a specific CDS on the array, and the CDSs are arranged in the order of the genome of strain 8004. Each green line indicates an AHD CDS in the corresponding test strain. The serial numbers on the right indicate the variable genomic regions of *Xcc*.

Using the parameters established above, the gene composition of 18 Chinese *Xcc *strains was analyzed by aCGH using the genome of strain 8004 as the reference. The results are shown in Tables 4 and 5, Figure 2 and Additional data file 4. Of the 4,186 CDSs spotted on the microarray slides, 3,405 are conserved in all of the strains tested (Table 5). These conserved CDSs may represent the common backbone ('core' genes) of the *Xcc *genome, which contains most of the genes encoding essential metabolic, biosynthetic, cellular, and regulatory functions (Table 5). The genes relevant to central intermediary metabolism, replication, transcription, translation, the TCA cycle, and nucleotide, fatty acid and phospholipid metabolism are largely conserved. Genes encoding the components involved in the type I (T1SS), type II (T2SS) and type III secretion systems (T1SS-T3SS) as well as extracellular polysaccharide production, and the *rpf *(regulation of pathogenicity factors) gene cluster [11,12] are highly conserved among the *Xcc *strains investigated, although some predicted pathogenicity- and adaptation-related genes are AHD (Table 5).

**Table 4:** The number of conserved and absent/highly divergent CDSs in *Xcc *strains

*Xcc *strains	CDSs annotated	CDSs on chip	Conserved CDSs	AHD CDSs*	Invalid
8004	4,273	4,186			6
CN01			3,905	270	11
CN02			3,821	361	4
CN03			3,888	294	4
CN04			3,806	376	4
CN05			3,921	261	4
CN06			3,771	374	41
CN07			4,045	137	4
CN08			3,870	310	6
CN09			3,930	252	4
CN10			3,937	245	4
CN11			3,916	265	5
CN12			3,846	335	5
CN14			3,706	475	5
CN15			3,812	370	4
CN16			3,809	373	4
CN17			3,774	406	6
CN18			3,809	372	5
CN20			3,914	268	4

*Altogether, 730 CDSs were AHD among the Chinese strains, of which 58 were commonly AHD in all the Chinese strains. Fifty-one CDSs were found to be given invalid results.

**Table 5:** Distribution of strain 8004's CDSs and the AHD CDSs by functional categories

Functional category	Annotated	Spotted	Conserved	AHD	Invalid	ADHs/spotted
C01 Amino acid biosynthesis	115	115	97	16	2	13.91%
C02 Biosynthesis of cofactors, prosthetic groups, carriers	114	113	107	3	3	2.65%
C03 Cell envelope and cell structure	167	165	136	26	3	15.76%
C04 Cellular processes	127	127	110	16	1	12.60%
C05 Central intermediary metabolism	185	184	164	16	4	8.70%
C06 Energy and carbon metabolism	214	214	189	20	5	9.35%
C07 Fatty acid and phospholipid metabolism	80	80	74	4	2	5.00%
C08 Nucleotide metabolism	52	52	48	4	0	7.69%
C09 Regulatory functions	260	260	220	36	4	13.85%
C10 Replication and DNA metabolism	139	139	112	25	2	17.99%
C11 Transport	257	257	226	30	1	11.67%
C12 Translation	254	253	235	18	0	7.11%
C13 Transcription	53	53	45	8	0	15.09%
C14 Mobile genetic elements	138	65	10	53	2	81.54%
C15 Putative pathogenicity factors	305	304	258	46	0	15.13%
C15.01 Type I secretion system	4	4	4	0	0	0.00%
C15.02 Type II secretion system	24	24	22	2	0	8.33%
C15.03 Type III secretion system	27	27	27	0	0	0.00%
C15.04 Type IV secretion system	19	19	5	14	0	73.68%
C15.05 Type V secretion system	4	4	4	0	0	0.00%
C15.06 Sec and TAT system	19	19	18	1	0	5.26%
C15.07 Type III-effectors and candidates	16	16	8	8	0	50.00%
C15.08 Host cell wall degrading enzymes	34	33	32	1	0	3.03%
C15.09 Exopolysaccharides	14	14	14	0	0	0.00%
C15.10 Lipopolysaccharides	29	29	21	8	0	27.59%
C15.11 Detoxification	44	44	43	1	0	2.27%
C15.12 Toxin and adhesin	14	14	10	4	0	28.57%
C15.13 Quorum sensing	26	26	25	1	0	3.85%
C15.14 Other pathogenicity factors	31	31	25	6	0	19.35%
C16 Stress adaptation	102	102	92	10	0	9.80%
C17 Undefined category	130	130	101	27	2	20.77%
C18 Hypothetical proteins	1,581	1,573	1,181	372	20	23.65%
Total	4,273	4,186	3,405	730	51	17.44%

The aCGH results showed that 730 CDSs are absent or highly divergent among all the Chinese strains tested (Tables 4 and 5). In addition, a total of 51 invalid hybridization spots (CDSs) were observed in all the aCGH analyses of the 18 Chinese strains. The 730 AHD genes, which account for 17.6% of all valid hybridized CDSs in the aCGH analyses, may constitute the *Xcc *flexible gene pool. The functional categories of all the AHD genes are given in Table 5. Half of the AHD genes have been predicted to encode proteins with unknown function.

The differences in the numbers of the AHD genes in different strains are significant (Table 4). Compared with the reference strain 8004, the most divergent Chinese *Xcc *strain is CN14, of which 475 CDSs are AHD; and the most closely related strain is CN07, of which only 137 CDSs are AHD. Fifty-seven *Xcc *8004 CDSs, most of them encoding hypothetical proteins, are AHD in all eighteen Chinese strains. Of the 57 CDSs, 16 are conserved in strain ATCC33913. A hierarchical clustering program [45] was used to explore the relationship of the different *Xcc *strains based on the aCGH analysis (Figure 2). The result shows that the Chinese strains and the reference strain are divided into five groups (Figure 2). Some *Xcc *strains classified in the same phylogenetic group based on 16S-23S rDNA ITSs showed a similar grouping pattern in hierarchical clustering (Figure 2 and Additional data file 2). However, no significant relationship was observed between phylogenetic group and pathogenicity, or pathogenicity and hierarchical cluster.

No significant correlations were observed between the gross genome composition of *Xcc *strains and their pathogenicity, or the genome composition of the strains and their geographical origins. However, strains CN14, CN15, and CN16, which were isolated from different host plants around Guilin city, are significantly conserved in genome composition and exhibit similar pathogenicity (Tables 1 and 2; Additional data file 4). This suggests that the three strains may share a most recent common ancestor that is different from that (those) of the other Chinese strains.

### The variable genomic regions and their divergence in different strains

The locations of the variable genes in the different strains identified by the aCGH analysis were mapped onto the chromosome of strain 8004. The results revealed that there are 27 such chromosomal regions, each of which consists of more than three contiguous CDSs in the 8004 genome (Figure 2). These regions were named XVRs for *Xanthomonas *variable genomic regions and numbered from 1 to 27 in accordance with the genome coordinates of strain 8004 (Table 6). The boundaries of the XVRs were determined at the CDS level, to fit in with the resolution of the array hybridization analysis in this study. The 27 XVRs contain 402 CDSs and account for 48.4% of the AHD genes, representing 9.41% of the total CDSs of *Xcc *strain 8004.

**Table 6:** The variable genomic regions in strain 8004

XVR	Chromosomal coordinates	CDSs	Length	GC	δ* value (×1,000)
XVR01	76036-80668	XC0061-XC0065 (5)	4,633	54.31	98.553
XVR02^†^	159333-170981	XC0128-XC0136 (8)	11,649	57.19	62.146
XVR03	269007-274301	XC0223-XC0225 (3)	5,295	57.89	51.416
XVR04	402049-414813	XC0341-XC0355 (15)	12,765	56.94	103.820
XVR05	562624-571104	XC0475-XC0480 (6)	8,481	55.55	101.685
XVR06	705062-714579	XC0589-XC0596 (8)	9,518	59.74	57.941
XVR07	1035995-1049889	XC0856-XC0871 (15)	13,895	57.67	56.843
XVR08	1095226-1097524	XC0914-XC0916 (3)	2,299	67.76	100.000
XVR09	1231170-1259018	XC1018-XC1042 (22)	27,849	55.22	91.389
XVR10	1270957-1275001	XC1055-XC1059 (5)	4,045	55.16	100.665
XVR11	1940629-1952343	XC1619-XC1626 (8)	11,715	56.06	80.616
XVR12	1958257-1968956	XC1631-XC1641 (11)	10,700	54.1	72.784
XVR13	2414668-2513025	XC2002-XC2089 (81)	98,358	60.51	77.060
XVR13.1^‡^	2432933-2490940	XC2020-XC2074 (53)	58,007	62.23	63.589
XVR14	2531325-2543429	XC2106-XC2126 (21)	12,105	60.05	46.543
XVR15	2545133-2569438	XC2128-XC2140 (13)	24,305	63.27	31.640
XVR16	2713064-2720842	XC2254-XC2258 (5)	7,779	64.51	55.837
XVR17^†^	2759130-2764563	XC2292-XC2295 (4)	5,434	59.26	65.992
XVR18^†^	2899536-2958586	XC2399-XC2444 (47)	59,051	55.38	109.955
XVR19	3122997-3176917	XC2590-XC2638 (49)	53,921	58.17	113.835
XVR20^†^	3332308-3356903	XC2774-XC2790 (17)	24,596	58.49	83.425
XVR21	3620451-3629704	XC3026-XC3034 (9)	9,254	61.49	40.074
XVR22	3809655-3818302	XC3180-XC3184 (5)	8,648	58.43	85.752
XVR23	4299842-4315783	XC3619-XC3633 (14)	15,942	56.82	81.182
XVR24	4382229-4384007	XC3695-XC3697 (3)	1,778	49.59	108.993
XVR25	4492839-4498618	XC3799-XC3804 (6)	5,780	57.77	73.882
XVR26	4614209-4631109	XC3908-XC3924 (16)	16,901	58.23	52.313
XVR27^†^	5009127-5011690	XC4232-XC4234 (3)	2,564	55.97	104.713

^†^These variable genomic regions (XVRs) are totally absent from the genome of Chinese strains. ^‡^XVR13.1 denotes that the fragment is a part of XVR13.

The size of the XVRs ranges from 1,778 bp (XVR24 with only three CDSs) to 98,358 bp (XVR13 with 81 CDSs) (Table 6). There are 15 XVRs larger than 10 kb and 4 larger than 50 kb. Within the XVRs, there are 27 genes encoding proteins for pathogenicity and adaptation, 9 for regulatory functions, 25 for cell structure and cell processes, 19 for intermediary metabolisms, 95 for mobile elements, 21 for DNA metabolism related to mobile elements, and 219 encoding hypothetical or function-unknown proteins (Table 6 and 7).

**Table 7:** The characteristics of the variable genomic regions in strain 8004

XVR	Sequence characteristics	Functional description	Occurrence of XVRs*
XVR01	Gene phage related	Regulatory protein cII, putative secreted proteins	II
XVR02	IS elements	Deoxycytidylate deaminase and Rhs protein, genes related T4 phage	I
XVR03	Gene phage related	Methyltransferase	III
XVR04	Integron	Integron, xanthomonadin biosynthesis	III
XVR05	Gene phage related	Type I site-specific deoxyribonuclease	II
XVR06	IS elements	ThiJ/PfpI family protein, oxidoreductase	II
XVR07	IS elements	VirB6 protein	II
XVR08		Transcriptional regulator BlaI family	III
XVR09	Integrase + tRNA-Gly IS elements	Regulatory protein BphR	II
XVR10		Fimbrial assembly protein	III
XVR11		Type IV pilin	III
XVR12	IS elements	VirB cluster for T4SS	III
XVR13	Integrase + tRNA-Gly IS elements	Avirulence proteins, pathogenicity related proteins	II
XVR13.1	IS elements Gene phage related	Adaptation, virulence related protein	II
XVR14	Gene phage related	Prophage	II
XVR15	IS elements	Histidine kinase/response regulator hybrid protein, single-domain response regulator	II
XVR16		Nucleotide sugar transaminase	III
XVR17	IS elements	Arsenite efflux, iron uptake	I
XVR18	Integrase + tRNA-Arg IS elements	Plasmid mobilization protein, hemolysin activation protein	I
XVR19	Integrase + tRNA-Ser IS elements	Avirulence protein, phage related protein	II
XVR20	IS elements Integrase	Phage related protein, helicase	I
XVR21		Metabolic enzymes	II
XVR22		Type I site-specific restriction-modification system, virulence protein	III
XVR23	IS elements	Sugar translocase, O-antigen	IV
XVR24	Flanked by IS elements		II
XVR25	IS elements	Avirulence protein, regulators	II
XVR26	IS elements Gene phage related	Rich in mobile elements	II
XVR27	IS elements	*Xmn*I methyltransferase	I

*There are four possible occurrences of the XVRs predicted in the *Xcc *genomes: I, recent horizontally acquired sequences; II, horizontally acquired sequences; III, inherited from a common ancestor of xanthomonads and lost from certain *Xcc *strains at a later stage; IV, inherited from a common ancestor of xanthomonads and degenerated in certain *Xcc *strains at a later stage.

The distribution patterns of XVRs show significant diversity among the *Xcc *strains tested (Table 8). Five XVRs (XVR02, XVR17, XVR18, XVR20 and XVR27) are AHD from all the Chinese strains tested (Table 8). XVR17 and XVR18 are also absent from the British strain ATCC33913 as pointed out by Qian *et al*. [21]. Most of the genes in these five XVRs encode hypothetical proteins for which there are no significantly similar sequences in GenBank.

**Table 8:** The distribution of variable genomic regions in *Xcc *strains

	Strains																		
	
XVR	33913	CN01	CN02	CN03	CN04	CN05	CN06	CN07	CN08	CN09	CN10	CN11	CN12	CN14	CN15	CN16	CN17	CN18	CN20
XVR01	+	-	-	-	-	-	-	+	-	-	-	-	-	-	-	-	-	-	+
XVR02	+	-	-	-	-	-	-	-	-	-	-	-	-	-	-	-	-	-	-
XVR03	+	-	-	-	-	+	-	+	-	-	-	-	-	+	+	+	-	-	-
XVR04	+	(-)	(-)	(-)	-	-	-	+	-	+	+	(-)	(-)	-	-	-	-	-	(-)
XVR05	+	-	-	-	-	+	-	+	-	+	+	-	-	-	-	-	-	-	(+)
XVR06	+	+	-	(+)	-	(+)	-	+	-	+	+	+	-	(+)	(+)	(+)	-	-	(+)
XVR07	+	-	-	-	-	-	-	(+)	-	+	+	-	-	-	-	-	-	-	(+)
XVR08	+	-	-	-	-	+	-	+	-	+	+	-	-	+	+	+	-	-	+
XVR09	+	-	-	-	-	+	-	+	-	+	+	-	-	-	-	-	-	-	+
XVR10	+	-	-	-	-	-	-	+	-	+	+	-	-	-	-	-	-	-	+
XVR11	+	-	-	-	-	+	-	+	-	+	+	-	-	-	-	-	-	-	+
XVR12	+	-	-	-	-	+	-	+	-	+	+	-	-	-	-	-	-	-	+
XVR13	(+)	+	-	+	-	(+)	-	(+)	-	(+)	(+)	+	-	-	-	-	-	-	(+)
XVR13.1	-	+	-	+	-	-	-	-	-	-	-	+	-	-	-	-	-	-	-
XVR14	+	-	-	-	-	+	-	+	-	-	-	+	-	-	-	-	-	-	+
XVR15	+	+	+	+	+	+	+	+	+	+	+	+	+	+	-	-	-	+	+
XVR16	+	+	-	+	-	+	-	+	-	+	+	+	-	+	+	+	-	-	+
XVR17	-	-	-	-	-	-	-	-	-	-	-	-	-	-	-	-	-	-	-
XVR18	-	-	-	-	-	-	-	-	-	-	-	-	-	-	-	-	-	-	-
XVR19	+	-	-	-	-	-	-	+	-	-	-	-	-	-	-	-	-	-	-
XVR20	+	-	-	-	-	-	-	-	-	-	-	-	-	-	-	-	-	-	-
XVR21	+	+	+	+	+	+	+	+	+	+	+	+	+	-	-	-	+	+	+
XVR22	-	+	+	-	+	-	-	+	+	+	+	+	+	-	+	+	+	+	+
XVR23	+	-	+	+	+	-	+	+	+	-	-	-	+	+	+	+	+	+	+
XVR24	+	+	-	-	-	+	-	+	-	+	+	+	-	-	-	-	-	-	+
XVR25	+	-	+	+	+	-	+	+	+	+	+	-	+	-	-	-	+	+	+
XVR26	+	+	-	+	-	+	-	+	-	-	-	+	-	-	-	-	-	-	+
XVR27	+	-	-	-	-	-	-	-	-	-	-	-	-	-	-	-	-	-	-

+, the XVR is present; -, AHD; (+), some CDSs of the XVR might be present and are ordered in the allele in the given genome; (-), a few CDSs of the XVR are scattered in the allele in the given genome.

XVR04 is a typical integron, which contains a gene for a DNA integrase (*intI*) catalyzing the site-specific recombination of gene cassettes at the integron-associated recombination site (*attI*), and a cassette array of 14 genes with unknown function [21,46]. Integrons are best known for assembling antibiotic resistance genes in clinical bacteria. They capture genes by integrase-mediated site-specific recombination of mobile gene cassettes. It has been postulated that the ancestral xanthomonad possessed an integron at *ilvD*, an acid dehydratase gene flanking the *intI *site-specific recombinase [46]. The microarray results showed that all of the Chinese strains tested possess the *ilvD *gene, although whether its organization is conserved in these strains is unknown. However, significant diversity found in the integron cassette array among these Chinese strains suggests that the integron might also generate diversity within the pathovar, in addition to between pathovars [46].

**Figure 3:**
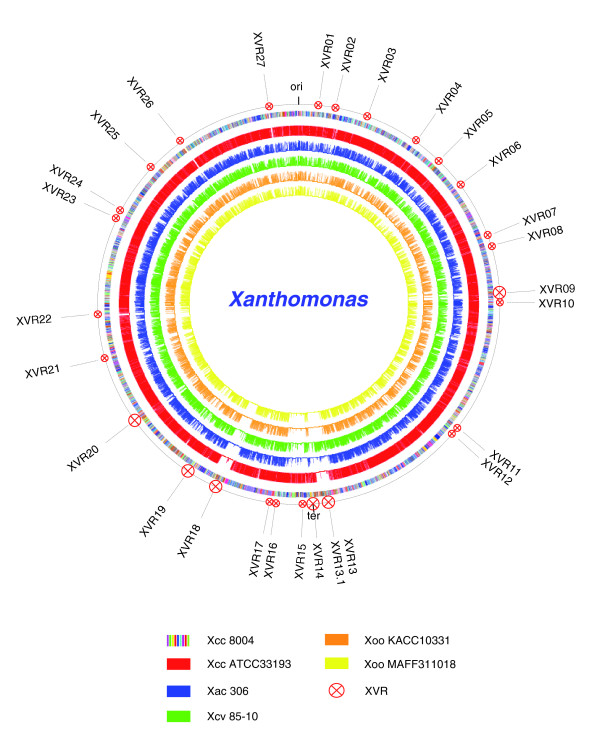
Whole genome comparison of the CDS set of strain 8004 with that of each sequenced xanthomonad strain. The circles display, from outside in: 1, the position of XVRs in the genome of *Xcc *8004; 2, the circular representation of genome of *Xcc *8004 (CP000050), map scaled in CDS; 3-7, BLASTN results of the CDS set of *Xcc *8004 with that of each sequenced xanthomonad strain, *Xcc *ATCC33913 (AE008922), *Xac *306 (AE008923), *Xcv *85-10 (AM039948), *Xoo *KACC10331 (AE013598), *Xoo *MAFF311018 (NC_007705).

XVR14 contains 21 CDSs with two copies of the phi Lf-like *Xanthomonas *prophage, which harbors the putative *dif *site of replication termination of the *Xcc *strains 8004 [21] and Xc17 [47]. In strain ATCC33913, the two copies of Lf-like prophage possess the typical genetic organization of filamentous phages, that is, a symmetrical head-to-head constellation, with genes functioning in DNA replication, coat synthesis, morphogenesis and phage export [20]. In strain 8004, only one copy of the Lf-like prophage is intact and the other lacks two genes (*gII *and *gV*) [20,21]. This phi Lf-like prophage is missing from or highly divergent in most of the Chinese strains tested and most other xanthomonads sequenced, but present in *Xcv *85-10 [48] (Table 9 and Figure 3). It is worth mentioning that the P2-like prophage [49], which occurs in strain ATCC33913 but is missing from strain 8004, is found to be AHD from all of the Chinese strains tested by hybridization analysis using a probe from ATCC33913 [20,21].

**Table 9:** The distribution of variable genomic regions in other sequenced *Xanthomonas *spp.

XVR	*Xac *306	*Xcv *85-10	*Xoo *KACC10331	*Xoo *MAFF311018
XVR01	(-)	-	-	-
XVR02	(-)	(-)	(-)	(-)
XVR03	(+)	(+)	+	+
XVR04	-	-	-	-
XVR05	-	+	-	-
XVR06	(-)	(-)	(-)	(-)
XVR07	(-)	(-)	-	-
XVR08	+	+	+	+
XVR09	(-)	(-)	-	-
XVR10	+	+	+	+
XVR11	+	+	+	+
XVR12	+	+	-	-
XVR13	(-)	(-)	-	-
XVR13.1	(-)	(-)	-	-
XVR14	-	(+)	-	(-)
XVR15	(-)	(-)	(-)	(-)
XVR16	(+)	(+)	(+)	(+)
XVR17	-	-	-	-
XVR18	-	-	-	-
XVR19	(+)	(+)	-	-
XVR20	(-)	(-)	(-)	(-)
XVR21	-	-	-	-
XVR22	(-)	+	-	-
XVR23	(-)	(-)	(-)	(-)
XVR24	(-)	(-)	(-)	(-)
XVR25	(-)	-	-	-
XVR26	(-)	(-)	(-)	(-)
XVR27	-	-	-	-

* Whole genome comparison results are given. +, the XVR is present; -, absent; (+), some CDSs of the XVR might be present and are ordered in the allele in the given genome; (-), a few CDSs of the XVR are scattered in the allele in the given genome.

There are two clusters of the type I restriction-modification system in strain 8004, of which one is present in strain ATCC33193 and the other is unique to strain 8004 [20,21]. XVR22 is one of these clusters. In contrast to ATCC33913, which lacks this locus, most of the Chinese strains possess it. Restriction and modification systems are responsible for cellular protection and maintenance of genetic materials against invasion of exogenous DNA. There is evidence that they have undergone extensive horizontal transfer between genomes, as inferred from their sequence homology, codon usage bias and GC content difference. In addition to often being linked with mobile genetic elements, such as plasmids, viruses, transposons and integrons, restriction-modification system genes themselves behave as mobile elements and cause genome rearrangements [50].

XVR23 consists of 14 ORFs that contains several genes for lipopolysaccharide (LPS) O-antigen synthesis, including *wxcC*, *wxcM*, *wxcN*, *gmd *and *rmd *[19], which is discussed below. Some predicted functions of other XVRs are shown in Table 7 based on the annotation of their component CDSs.

### Horizontal gene acquisition and gene loss

The detection of DNA segments in which integrase genes are associated with tRNA or tmRNA genes [51-53], or regions of anomalous GC content with mobile elements [54], facilitates the identification of horizontally acquired sequences in genomes. Horizontally acquired sequences are also detectable by comparing their dinucleotide composition (genome signature) dissimilarity (δ* value) with that of the host genome. The higher δ* values of XVRs can be indicative for horizontal acquisition [55]. The data presented in Tables 6 and 7 show that XVR09, XVR13, XVR18 and XVR19 are integrated adjacent to or within tRNA genes with an integrase or insertion sequence (IS) flanking the ends. XVR04, an integron [46], and XVR14, a phi Lf-like prophage [20,21], are also actively transferred DNA sequences. Obviously, the five XVRs, XVR02, XVR17, XVR18, XVR20 and XVR27, which are ubiquitously AHD from all the Chinese strains tested, could be the most recently acquired DNA in strain 8004. It is possible that the donors of these five XVRs are probably absent in mainland China. In contrast, we consider that the XVRs present in the other sequenced xanthomonad strains may be a result of acquisition events during the early stage of *Xanthomonas *evolution and lost from certain *Xcc *strains at a later stage, probably due to DNA deletion events.

The identification of *Xcc *DNA loss events can be carried out by analysis of the sequenced xanthomonads for the presence of collinear blocks that encompass the targeted DNA segments. Whole genome comparisons among *Xcc *8004 [21], *Xcc *ATCC33913 [20], *X. axonopodis *pv. *citri *306 [20], *X. campestris *pv. *vesicatoria *85-10 [48], *X. oryzae *pv. *oryzae *KACC10331 [56] and *X. oryzae *pv. *oryzae *MAFF311018 [57], allowed identification of a number of XVRs (XVR03, XVR05, XVR08, XVR10, XVR11 and XVR22) as DNA segments inherited from the common ancestral xanthomonad (Figure 3). In each case, large DNA segments containing each of these XVRs have a high degree of synteny in other xanthomonads (Figure 3 and Table 9).

**Figure 4:**
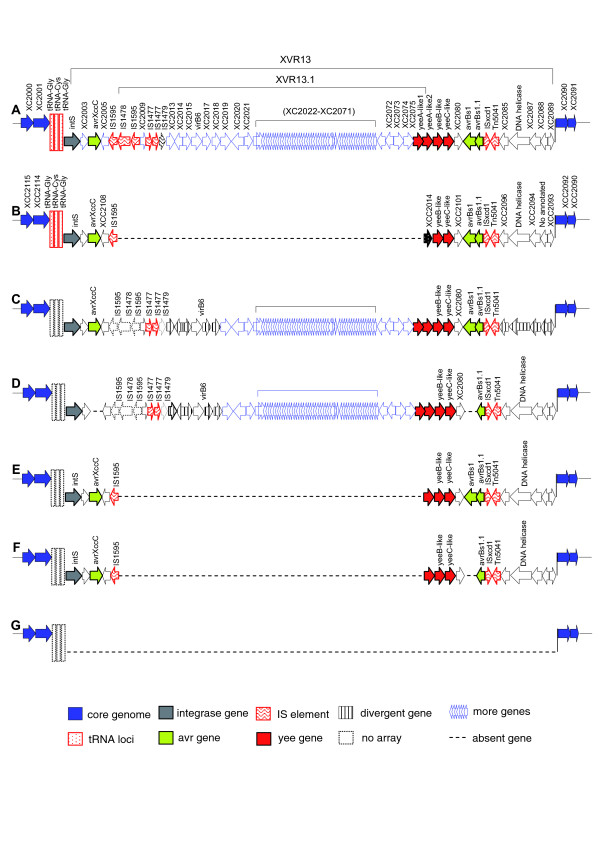
The presumed allelic loci of XVR13 in the Chinese *Xcc *strains suggested by aCGH. **(a) **The genomic region XVR13 in strain 8004. **(b) **The allelic locus of XVR13 in strain ATCC33913 revealed by whole genome comparison. **(c-h) **The allelic loci of XVR13 in the Chinese *Xcc *strains revealed by aCGH: (c) strains CN01, CN03 and CN11; (d) strains CN10 and CN20; (e) strain CN09; (e) strain CN07; (g) strains CN02, CN04, CN05, CN06, CN08, CN12, CN15, CN16 and CN18; (h) strains CN14 and CN17. IS, insertion sequence.

Analysis of the structure of XVR13 and its distribution pattern in *Xcc *strains revealed that this region might undergo a series of multiple insertion and deletion events during the *Xcc *evolution (Figure 4). This region is near the terminus of chromosome replication, which is susceptible to gene acquisition and/or gene loss [20]. XVR13 is the largest genomic island identified in *Xcc *8004, which spans nucleotide coordinates from 2,414,668 to 2,513,025 and contains 81 CDSs. To its left flank are three tRNA genes and an integrase gene. Genome comparison showed that the central part of XVR13, named XVR13.1, is totally absent in strain ATCC33913. XVR13.1 is 58,007 bp in length. The aCGH results reveal that three Chinese strains (CN01, CN03 and CN11) contain the XVR13 locus, which is almost identical to that of *Xcc *8004, and four Chinese strains (CN07, CN09, CN10 and CN20) contain an incomplete XVR13 locus without XVR13.1 that is almost identical to that in strain ATCC33913, and the rest of the Chinese strains probably have no XVR13 (Table 8 and Figure 4). To elucidate the dynamic relationship between XVR13 and XVR13.1, re-annotation was done for XVR13.1 and 63 CDSs were identified (Figure 4 and Additional data file 5). A truncated *yeeA*-like gene was found across the right border of XVR13.1 (Figure 4). Intriguingly, *yeeB- *and *yeeC*-like genes occur in both *Xcc *strains 8004 and ATCC33913 (Figure 4). This suggests that XVR13.1, or at least part of it, has been lost from the British strain ATCC33913 and most of the tested Chinese strains during their evolution.

XVR23, part of the *wxc *cluster, contains several genes for O-antigen synthesis of LPS [21]. The aCGH results revealed that this region is highly divergent, with a mosaic structure among the Chinese strains tested. Sequence comparisons showed that *wxc *cluster of *Xcc *8004 is significantly divergent from that of *Xcc *B100 [19], although it is almost identical to that of *Xcc *ATCC33913 [20,21]. The *wxc *cluster of strain 8004 is truncated by IS elements and some of the *wxc *genes have low similarity to the corresponding genes of strain B100. Significant differences in *wxc *clusters among other xanthomonad strains have also been reported [48,56,58]. The *Xcc wxc *cluster not only has a significantly lower GC content (56.82%) than the average genome level (64.95%), but also has a very high δ* value of 81.182. These suggest that *Xcc *might have acquired the *wxc *cluster by horizontal DNA transfer.

### The distribution of pathogenicity-related genes among *Xcc *strains

Bioinformatic analysis revealed that strain 8004 contains 197 CDSs that show homology to the confirmed or annotated putative pathogenicity genes of plant or animal pathogenic bacteria, in addition to 108 genes that have been proven to be involved in *Xcc *pathogenicity (Additional data file 6). Of these 305 proven or presumed pathogenicity genes, 304 were spotted on the microarray slides of strain 8004 in this study. The other CDS (*XC3591*) encoding pectate lyase was not spotted as it has a redundant DNA sequence in the genome of strain 8004. The aCGH analysis revealed that 258 of the pathogenicity genes (84.8% of the pathogenicity genes spotted) are present in all of the *Xcc *strains tested and 46 (15.1%) are AHD in at least one of the strains (Table 5 and Additional data file 6). The results show that the pathogenicity genes involved in the type I, II and III secretion systems (T1SS, T2SS and T3SS), host cell wall degradation, extracellular polysaccharide production, and the quorum sensing system are highly conserved in almost all of the *Xcc *strains tested (Table 5 and Additional data file 6). In addition, genes encoding proteins of the gluconeogenic pathway [59], Mip-like protein [60], the catabolite repressor-like protein Clp [61], and zinc uptake regulator protein Zur [44], which have been demonstrated to play important roles in *Xcc *virulence, are also highly conserved. However, genes relating to T4SS, T3SS-effectors and candidates, LPS synthesis, toxin as well as adhesin are highly diversified (Table 5 and Additional data file 6).

LPS is an indispensable component of the cell surface of Gram-negative bacteria and has been demonstrated to play important roles in pathogenicity of several phytopathogenic bacteria, including *Xcc *[62-64]. More than 20 genes for LPS synthesis have been characterized in *Xcc*. These include *xanAB *[65], *rmlABCD *[66], *rfaXY *[64], *lpsIJ *[67] and the *wxc *cluster consisting of 15 genes [19]. The aCGH results suggest that *lpsIJ*, *rfaXY*, *rmlABCD *and *xanAB *are highly conserved while *wxc *genes are divergent in the *Xcc *strains tested. The *wxc *genes are involved in the biosynthesis of the LPS O-antigen, which is the most variable portion of LPS [19,68]. The diversity of the *wxc *cluster indicates that the LPSs produced by *Xcc *different strains may be varied.

**Figure 5:**
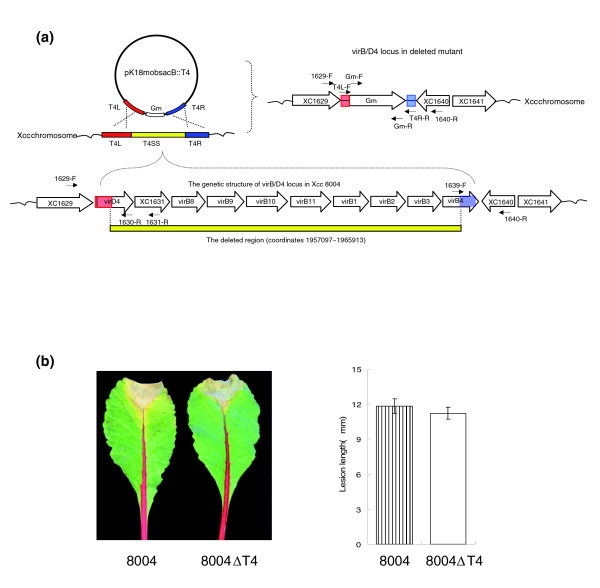
Analysis of the T4SS locus in *Xcc *8004. **(a) **The mutagenesis strategy used in the deletion of T4SS (*virB*/*D4 *locus). The red and blue rectangles indicate the T4SS left and right border sequences, respectively, which were cloned and used as the target sequences in homologous recombination. The yellow rectangle indicates the deleted region of the *virB*/*D4 *locus. The larger hollow arrows indicate the CDSs and the smaller solid arrows indicate the primers used. **(b) **The plant assay results of the wild-type strain 8004 and the mutant 8004ΔT4 on the host plant radish (*R. sativus *var. *radicula*) cv. Manshenhong. The photographs were taken ten days after inoculation. The average lesion lengths caused by the 8004ΔT4 mutant were not significantly different from those caused by the wild type. Values are the mean ± standard deviation from 3 repeats, each with 50 leaves.

T4SSs have been validated as having important roles in the pathogenesis of several animal and plant bacterial pathogens [36-38,40]. The T4SS of *Agrobacterium tumefaciens *is essential for virulence and is assembled from the proteins encoded by the *virB *cluster and *virD4*. Many T4SSs are highly similar to the *A. tumefaciens *VirB/D4 T4SS [40]. *Burkholderia cenocepacia *strain K56-2 can produce the plant tissue watersoaking phenotype (a plant disease-associated trait) and possesses two T4SSs similar to the VirB/D4 system [69]. Mutational studies in *B. cenocepacia *strain K56-2 revealed that the plasmid-encoded T4SS is involved in eliciting the plant tissue watersoaking phenotype and responsible for the secretion of a plant cytotoxic protein(s), while the chromosome-encoded T4SS is not [69]. Genome annotation revealed that the *Xcc *strain 8004 has an *A. tumefaciens *VirB/D4-like T4SS [21]. Although genomic sequence comparison showed that the *Xcc *strain ATCC33913 possesses an almost identical *virB *cluster to that of strain 8004, the aCGH analyses displayed that the *virB *cluster of most Chinese strains tested is AHD. Since all these strains were fully virulent and their aCGH intensity ratios were extremely low (as low as 0.1-0.025; Additional data file 4), a query on the role of the T4SS in *Xcc *pathogenicity was raised. To answer this question, we constructed a T4SS mutant derived from strain 8004 (Figure 5). A mutant with deletions of the *virB *cluster as well as *virD4 *was confirmed by PCR and designated 8004ΔT4 (Figure 5 and Additional file 7). The virulence of the mutant was tested on host plants cabbage (*B. oleracea *var. *capitata*) cv. Jingfeng-1, Chinese cabbage (*B. rapa *subsp. *pekinensis*) cv. Zhongbai-83, Chinese kale (*B. oleracea *var. *alboglabra*) cv. Xianggangbaihua, pakchoi cabbage (*B. rapa *subsp. *chinensis*) cv. Jinchengteai, and Radish (*R. sativus *var. *radicula*) cv. Manshenghong by the leaf-clipping inoculation and spray methods. The results showed that the virulence of the mutant was as severe as on the wild type strain 8004 on all the tested plants inoculated by leaf-clipping (Figure 5) or spray (data not shown). This suggests that the T4SS is not involved in the virulence of *Xcc*.

### The genetic determinants for host specificity of *Xcc*

Genes involved in the host specificity of *Xcc *are of central interest in this study. All of the *Xcc *strains used in this work are able to cause disease in their host plants but show specificity for a host range. Apart from four strains (CN01, CN04, CN05 and CN17) that could infect all of the host plants tested, the other 16 strains were avirulent on certain host plant(s) (Table 2). The host specificity of pathogens is determined by gene-for-gene interactions [10] involving avirulence (*avr*) genes of the pathogen and cognate resistance (*R*) genes of the host. Disease resistance occurs in a host-pathogen interaction in which an *R *gene in the host is matched by a cognate *avr *gene in the challenging pathogen. A pathogen-host interaction without such a cognate *avr*-*R *combination will lead to disease.

To elucidate the genetic determinants for host specificity of *Xcc*, the correlation between the virulence scale on host plants and the gene distribution pattern of the 20 *Xcc *strains was analyzed. The correlation between HR induction on non-host plants and gene distribution patterns of the strains was also determined. Twelve operations were performed and the correlation coefficient (CC) values of these are given in Additional data files 8 and 9. Seven of the eleven host plants are susceptible to all of the 20 *Xcc *strains tested (Table 2), indicating that they have no CC values. Correlation analyses for the other four host plants and one non-host plant discovered four candidate genes responsible for the virulence-deficiency (negative CC value) of *Xcc *strains on a particular host plant(s) and one candidate for HR induction (positive CC value) on the non-host plant pepper ECW10R. These genes are candidates of the three postulated *avr *genes *avrRc1*, *avrRc3 *and *avrRp1 *(Table 10). The candidates *XC2004 *and *XC2084 *are correlative to *avrRc3 *and have the same CC value. *XC2084 *encodes a transposase [21], suggesting that its postulated *avrRc3 *is much smaller than that of *XC2004*. Therefore, *XC2084 *was removed from the candidate list. The candidate genes *XC2602*, *XC2004 *as well as *XC2081 *have been annotated as encoding Avr-homologous proteins [21].

**Table 10:** Identification of the genetic determinants for host specificity of *Xcc *8004 on certain plants

Candidate *avr *genes in *Xcc *8004				Mutational analysis^†^			
		
Postulated *avr *gene	Gene ID	Annotated function	Correlation (CC)*	Mutant ID	Virulence	8004	Plants
*avrRc1*	*XC2004*	Avirulence protein, *avrXccC*	-1	NK2004	+	-	TP1
	*XC2084*	Tn5041 transposase	-1	...	...	...	...
*avrRc3*	*XC2602*	Avirulence protein, *avrXccE1*	-0.88	NK2602	+	-	TP8
*avrRp1*	*XC2081*	Avirulence protein, *avrBs1 *gene	1	NK2081	N	HR	TP12

*The direct correlation analysis between virulence of the 20 *Xcc *strains on one given plant cultivar and the distribution status of each gene in 20 *Xcc *strains was carried out using the program CORREL. The maximal absolute CC values were selected from Additional data file 8. ^†^The mutants of the candidate genes were generated by means of pK18*mob *integration and the plant tests were carried out on given plants that showed specific reactions to certain *Xcc *strains. TP1, mustard (*B. juncea *var. *megarrhiza *Tsen et Lee) cv. Guangtou; TP8, Chinese cabbage (*B. rapa *subsp. *pekinensis*) cv. Zhongbai-83; TP12, pepper (*Capsicum annuum *v. *latum*) ECW10R. +, virulent; -, non-pathogenic. The hypersensitive reaction (HR) tests of *Xcc *strains were carried out on TP12. HR, positive HR result; N, no HR. ..., data unavailable.

**Figure 6:**
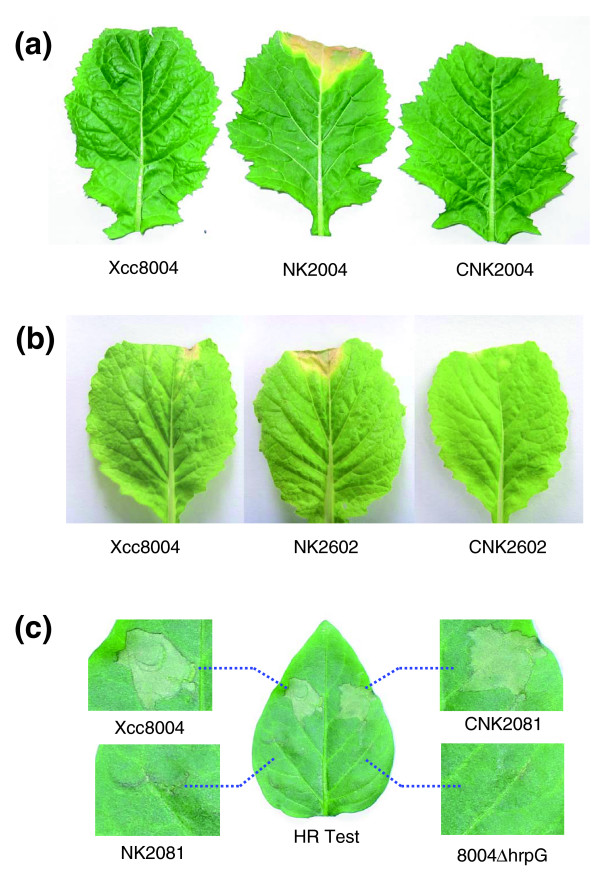
Virulence and HR of *Xcc *strains. **(a) ***Xcc *8004 and the complementary strain CNK2004 are avirulent, but the mutant NK2004 is virulent on the host plant, mustard (*B. juncea *var. *megarrhiza *Tsen et Lee) cv. Guangtou. The photographs were taken ten days after inoculation. **(b) ***Xcc *8004 and the complementary strain CNK2602 are avirulent, but the mutant NK2602 is virulent on the host plant, Chinese cabbage (*B. rapa *subsp. *pekinensis*) cv. Zhongbai-83. The photographs were taken ten days after inoculation. **(c) ***Xcc *8004 and the complementary strain CNK2081 could induce HR, but the mutant NK2602 and the negative control 8004Δ*hrpG *could not induce HR on the non-host plant, pepper (*Capsicum annuum*) cv. ECW10R. The photographs were taken 24 h after inoculation.

To identify *avr *genes from the candidates, we further investigated their biological functions by mutagenesis. The candidate *avr *genes of *Xcc *8004 were disrupted by using the plasmid pK18*mob *[70], a conjugative suicide plasmid in *Xcc *(see details in Materials and methods). The obtained nonpolar mutants of *XC2602*, *XC2004 *and *XC2081*, named NK2602, NK2004, and NK2081, respectively, were inoculated on corresponding host or non-host plants to test their virulence or HR. The results revealed that mutation in *XC2004 *or *XC2602 *altered the reaction of the pathogen on the corresponding host plant mustard cv. Guangtou or Chinese cabbage cv. Zhongbai-83, respectively, from non-pathogenic to pathogenic (Figure 6 and Table 10). Disruption of *XC2081 *resulted in the loss of the ability to elicit an HR on the non-host plant pepper ECW10R (Figure 6 and Table 10). These alterations in plant response caused by mutation in *XC2004*, *XC2602 *or *XC2081 *could be restored to the wild-type phenotype by expression *in trans *of the intact corresponding CDS carried by a DNA fragment cloned into pLAFR3 or pLAFR6 (Figure 6 and Table 10). These results demonstrate that *XC2004*, *XC2602 *and *XC2081 *are the postulated *avrRc1*, *avrRc3 *and *avrRp1*, respectively. *XC2004*, *XC2602 *and *XC2081 *of strain 8004 have been annotated as *avrXccC*, *avrXccE1 *and *avrBs1*, respectively, based on their sequence homology to *avr *genes identified in other pathogens [21]. Therefore, we renamed these postulated *avr *genes *avrRc1*, *avrRc3 *and *avrRp1 *as *avrXccC*, *avrXccE1 *and *avrBs1*, respectively (Table 10). Recently, Castañeda and associates [22] have shown that the avirulence of *Xcc *strain 528^T ^(*Xcc *ATCC33913) on Florida Mustard is attributed to *avrXccFM*, which shares the same locus as *avrXccC *but is longer than the *avrXccC *ORF annotated in the genome of ATCC33913 [20]. Our results further confirm that the *avrXccC *locus dominates the avirulence of *Xcc *on mustard plants. The avirulence function of *Xcc avrBs1 *is similar to that of the homologue *avrBs1 *of *Xcv *on the resistant pepper ECW10R, which contains the corresponding *R *gene *Bs1 *[71].

**Figure 7:**
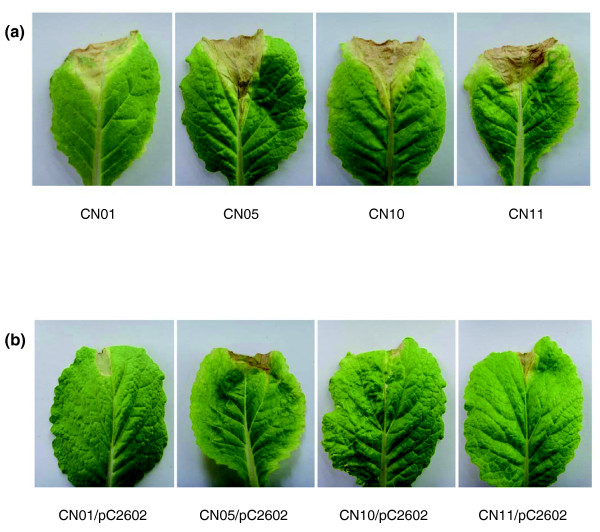
The intact *avrXccE1 *CDS of *Xcc *8004 confers avirulence function on the host plant Chinese cabbage (*B. rapa *subsp. *pekinensis*) cv. Zhongbai-83 to other *Xcc *strains. **(a) **The black rot symptoms on Chinese cabbage cv. Zhongbai-83 infected by the strains CN01, CN05, CN10 and CN11. The *avrXccE1*-allelic loci of these strains are AHD. **(b) **The strains CN01, CN05, CN10 and CN11 harboring the plasmid pC2602 (pLAFR6 carrying the intact *avrXccE1 *gene) show incompatible reaction on Chinese cabbage cv. Zhongbai-83. The photographs were taken ten days after inoculation.

To verify the avirulence function of *Xcc avrXccE1 *(*XC2602*), the cosmid pLAFR6 carrying a PCR-generated 1,605 bp fragment encompassing the region 514 bp upstream of the start codon to 29 bp downstream of the stop codon of *XC2602 *was introduced by triparental mating into the Chinese strains CN01, CN05, CN10 and CN11, which showed virulence on Chinese cabbage cv. Zhongbai-83 (Table 2). The obtained transconjugants for all the four strains lost virulence on Chinese cabbage cv. Zhongbai-83 (Figure 7). These results demonstrate that *avrXccE1 *(*XC2602*) of *Xcc *is endowed with an *avr *function determining host specificity.

## Discussion

In this work, we constructed a whole-genome microarray based on the determined genome sequence of *Xcc *strain 8004 isolated in the UK and used it to explore by aCGH analyses the genome contents and gene diversity among 18 *Xcc *strains isolated from different host plants and various geographical regions over a wide range of latitudes across China. Several attractive divergent genetic determinants related to pathogenicity uncovered by aCGH analyses were further functionally characterized, enabling the discovery of *avr *genes affecting *Xcc *host specificity and the T4SS that are not involved in symptom production by *Xcc*.

Our aCGH analyses revealed that 3,405 (81.3%) of the 4,186 genes of the *Xcc *strain 8004 spotted on the array were conserved in all the 18 Chinese *Xcc *strains tested. These conserved genes represent a rough genetic core of *Xcc*. This percentage is much higher than the 53% observed in 17 strains of the phytopathogenic bacterium *Ralstonia solanacearum *[35]. The *Xcc *core content contains not only the genes for essential metabolism, but also the genes encoding the main pathogenicity factors (see below) and proteins involved in xanthomonadin biosynthesis. The aCGH analyses also revealed that the *Xcc *strains possess a flexible gene pool of 730 CDSs, accounting for 17.6% of all the valid hybridized 4,135 CDSs in the aCGH analyses of all 18 Chinese strains. These genes are AHD from the Chinese strains compared with the reference strain 8004. The number of AHD CDSs of individual strains ranges from 137 to 475, which is more than the 108 strain-specific genes of *Xcc *8004 compared to strain ATCC33913 and the 62 strain-specific genes of ATCC33913 compared to *Xcc *8004, revealed by comparison of the two strains' whole genome sequences [20,21]. Among the 730 flexible genes, 58 are AHD from all the Chinese strains. Of these, 57 are situated in eight XVRs while one is alone; 42 located mainly in XVR13.1, XVR17 and XVR18 are also absent from the British strain ATCC33913 [21]. Whether the remaining 16 ADH CDSs in XVR02, XVR14, XVR20, XVR23 and XVR27, which are conserved in the British strains 8004 and ATCC33913, constitute the major genetic differences between British *Xcc *strains and Chinese *Xcc *strains needs further studies on more strains. Most of the 27 XVRs possess DNA sequences associated with integrase genes or mobile elements and with lower GC content and higher δ* value compared to *Xcc *regular genomic characteristics, implying that these DNA sequences may have been acquired through horizontal gene transfer [53,72].

Since all the strains used in this study are fully virulent in certain host plants, the genetic core revealed by aCGH characterization of these strains should cover the pathogen's symptom production and the basic pathogenicity determinants of the pathogen; hence the flexible genes might not be essential for virulence of the pathogen. The leaf-clipping inoculation method used for the pathogenicity tests in this study directly delivers bacterial cells into the vascular system of the host plant. Some of the genes involved in the early stages of the interaction between the pathogen and the host might be concealed in the flexible gene pool.

Eight *avr *genes are annotated in the genome of both *Xcc *strains 8004 and ATCC33913 based on their sequence homology to *avr *genes identified in other pathogens [20,21]. It has been shown that mutagenesis of all the eight *avr *genes in *Xcc *strain 528^T ^(ATCC33913) has no detected effect on virulence and only one of the *avr *genes affects race specificity [22]. However, it has been proposed that *Xcc *is composed of 6 races, based on the interactions of 144 isolates with 6 different host varieties in the 4 *Brassica *species *B. carinata*, *B. juncea*, *B. oleracea *and *B. rapa *[73]. The 20 strains used in this study could be grouped into three races based on their disease reactions on nine host varieties (or subspecies) in three *Brassica *species, *B. junccea*, *B. oleracea *and *B. rapa*, as well as the *Raphanus *species *R. sativus*. In addition, eight strains, including 8004 and ATCC33913, could induce HR on the non-host plant pepper ECW10R carrying the *R *gene *Bs1 *[71], indicating that these strains harbor a cognate *avr *gene *avrBs1*. To identify the *avr *genes in *Xcc *strain 8004, we employed a correlation analysis between the strain-plant reaction and the gene distribution pattern of strains to screen *avr *candidates and then ascertained the avirulence function of the candidates by genetic experiments. This strategy allowed us to identify the *avr *genes *avrXccC*, *avrXccE1 *and *avrBs1*. The *avrXccC *gene of strain 8004, conferring avirulence on mustard cultivar Guangtou, is identical with the *avrXccFM *of strain 528^T ^(ATCC33913), conferring avirulence on Florida mustard [22]. This study verified that *avrXccE1 *affects host specificity by conferring avirulence on Chinese cabbage cv. Zhongbai-83. The *avrXccE1 *of strain 8004 is identical to the *XCC1629 *of strain 528^T^. These two strains showed incompatible reactions on Chinese cabbage cv. Zhongbai-83 (Table 2). Castañeda and associates [22] did not observe such an avirulence function for *XCC1629 *of strain 528^T ^on Early Jersey Wakefield cabbage, suggesting that the *R *gene responsive to *avrXccE1 *(*XCC1629*) exists in Chinese cabbage cv. Zhongbai-83 but not in Early Jersey Wakefield cabbage. The *avrBs1 *of strain 8004 was validated to be responsible for eliciting HR on the non-host plant pepper ECW10R. The sequences of *avrBs1 *of strain 8004 and *XCC2100 *of strain 528^T ^(ATCC33913) are exactly the same [20,21]. Both strains could induce HR on the pepper ECW10R (Table 2) [22]. However, Castañeda and associates did not detect HR variation between the *XCC2100 *mutants and the wild-type 528^T ^[22]. It is possible that the function of *avrBs1 *is redundantly encoded in 528^T ^and that the expression and regulation of *avrBs1 *and *XCC2100 *in 8004 and 528^T ^(ATCC33913) is different. The postulated *avr *gene *avrRc2 *exists in the strains ATCC33913, CN14, CN15 and CN16 but not in the aCGH reference strain 8004. Work to identify *avrRc2 *from the ATCC33913-strain specific CDSs (compared to strain 8004) are underway.

Avirulence genes have been generally identified by molecular genetic methods where clones from a genomic library of an avirulent strain are mobilized into a virulent strain and the resulting transformants or transconjugants are tested for an alteration in the outcome of the pathogen-host interaction [74-77]. Genomic mining has also provided a powerful tool to uncover *avr *genes by homology searches and bioinformatic approaches [78-80]. Comparatively, a major advantage of the aCGH approach in identifying host specificity genes is the high-throughput and efficiency at identifying genome diversity at the gene level. This allows parallel identification of candidate genes for a number of avirulence determinants through the correlation analysis between the phenotype (avirulence/virulence) and the gene distribution pattern in a bacterial strain population. It could be expected that analysis of an increased number of strains in parallel with virulence assays on an increased number of host plants will enhance a full-scale identification of host specificity genes from a pathogen. The main limitations of the current aCGH approach are that it satisfies only the analysis of genes present in the reference strain and that it is incapable of identifying single nucleotide polymorphisms that may also contribute to gene functions.

Our aCGH results revealed that 258 (84.8%) of the 304 proven or presumed pathogenicity genes are conserved in all the *Xcc *strains tested and 46 (15.1%) are AHD. A large portion of these AHD genes are the *wxc *genes and the genes encoding T4SS as well as T3SS-effectors. The *wxc *gene cluster is involved in the synthesis of the LPS O-antigen. In *Xcc*, LPS has been demonstrated to play important roles in pathogenicity [64] and disruption of the *wxc *genes resulted in significant reduction of virulence [21]. As all the *Xcc *strains used in this study are fully virulent at least on some of the host plants tested, these strains may not be defective in LPS production. The diversity of the *wxc *genes suggests that the LPSs synthesized by *Xcc *different strains may have different structures.

Among plant bacterial pathogens, the role of T4SS in pathogenicity has not been experimentally verified except in *A. tumefaciens *[37], although T4SSs have been annotated as putative pathogenicity-related machines in the genomes of many pathogens, including the *Xcc *strains ATCC33913 and 8004 [20,21]. The high divergence of T4SS among the virulent *Xcc *strains revealed by the aCGH analyses prompted us to validate the role of T4SS in *Xcc *pathogenicity by genetic experiments. The T4SS in strain 8004 is encoded mainly by *virD4 *and the *virB *cluster, which consists of nine ORFs [21]. Deletion of *virD4 *and the *virB *cluster of strain 8004 did not affect the virulence of the pathogen on all the host plants tested, indicating that the T4SS is not engaged in the pathogenicity of *Xcc*. What is the function of T4SS in *Xcc*? Is it involved in bacterial conjugation and/or effector translocation? This will be the subject of future studies. Genomic data show that an entire T4SS encoded by *virD4 *and the *virB *cluster also exists in the phytopathogenic bacteria *Erwinia carotovora *[81], *Pseudomonas syringae *pv. *phaseolicola *[82], *R. solanacearum *[83], *X. axonopodis *pv. *citri *[20], *X. campestris *pv. *vesicatoria *[48] and *X. fastidiosa *[84]. To investigate experimentally the role of the T4SS in the pathogenicity of these pathogens will no doubt facilitate the understanding of the T4SS functions in plant bacterial pathogenesis.

## Conclusion

The results of our aCGH analyses reveal that about 80% of CDSs (3,405 CDSs) are conserved among 20 different virulent strains of *Xcc*. These conserved CDSs may stand for the core genome of *Xcc*, although the variable genes will increase in quantity with more strains to be analyzed. The core genome includes not only house-keeping genes but also a large amount (258) of proven or presumed pathogenicity-related genes. This work has also demonstrated that the T4SS, which has been validated to play important roles in the pathogenesis of a number of animal and plant bacterial pathogens and predicted to be a pathogenicity-related machine in many bacterial genomic annotations, is not involved in the pathogenicity of *Xcc*. Compared to the reference strain 8004, the number of flexible genes of individual Chinese *Xcc *strains ranges from 137 to 475. The *wxc *gene cluster, which is involved in LPS O-antigen synthesis and the pathogenicity of *Xcc*, is highly divergent among different *Xcc *strains. It is possible that the LPSs synthesized by different *Xcc *strains have various structures. We show an efficient strategy to identify *avr *genes determining pathogens' host specificities. Three *avr *genes from the *Xcc *strain 8004 were identified by the application of this strategy in this study. More *avr *genes in the *Xcc *strain 8004, if present, could be discovered by this approach with more different host plants.

## Materials and methods

### Bacterial strains, culture conditions and molecular manipulations

*Xcc *isolates used in this study were collected from various geographical locations over a wide range of latitudes across mainland China (Tables 1 and 2). The bacteria were isolated from the infected leaves of cruciferous plants with typical symptoms of black rot disease. Recovered colonies were picked and re-streaked onto NYG [14] agar plates to verify the bacterial identity. Each isolate was inoculated onto radish (*R. sativus *var. *radicula*) cv. Manshenhong by the leaf clipping method [85] to evaluate its pathogenicity. The 16S-23S rDNA ITS was amplified as described by Gurtler and Stanisich [41] using primer R1 and primer R2 (Additional data file 1).

Molecular manipulations, genomic DNA preparations, restriction endonuclease digestions and PCR amplifications were performed as described by Sambrook *et al*. [86]. Enzymes were supplied by Promega (Shanghai, China) and used in accordance with the manufacturer's instructions.

### Plant assays

The virulence of *Xcc *strains was evaluated on 11 host plants: cabbage (*B. oleracea *var. *capitata*) cv. Jingfeng-1, Chinese cabbage (*B. rapa *subsp. *pekinensis*) cv. Zhongbai-4, Chinese cabbage (*B. rapa *subsp. *pekinensis*) cv. Zhongbai-83, Chinese kale (*B. oleracea *var. *alboglabra*) cv. Xianggangbaihua, kohlrabi (*B. oleracea *var. *gongylodes*) cv. Chunqiu, mustard (*B. juncea *var. *megarrhiza *Tsen et Lee) cv. Guangtou, pakchoi cabbage (*B. rapa *subsp. *chinensis*) cv. Jinchengteai, pakchoi cabbage (*B. rapa *subsp. *chinensis*) cv. Naibaicai, radish (*R. sativus *var. *sativus*) cv. Cherry Belle, radish (*R. sativus *var. *longipinnatus*) cv. Huaye, and radish (*R. sativus *var. *radicula*) cv. Manshenhong (Table 2). All of these cultivars are available from the Institute of Vegetables and Flowers, Chinese Academy of Agricultural Sciences, Beijing 100081. Each *Xcc *strain was tested on all the 11 cultivars. The bacteria grown overnight in NYG medium [14] were washed and resuspended in water to a cell density OD of 0.01 at 600 nm. The last completely expanded leaf of the five-week old seedlings was inoculated by cutting with scissors dipped in bacterial suspensions [85] or by spraying the bacterial suspensions with a sprayer. Twenty leaves were inoculated for each strain-plant combination. The inoculated plants were kept in a culture room at a temperature of 28°C and a relative humidity of 80% under 16 h light day, after 24 h moisture preservation in a plastic chamber at a temperature of 28°C and a relative humidity of about 100%. First symptoms appeared five days post-inoculation, and the lesion lengths of 20 leaves were measured 10 days post-inoculation for each strain-plant combination. The virulence of each *Xcc *strain on each host plant was rated according to the disease symptoms: non-pathogenic, leaves with no visible effect, or with localized necrosis (HR) or with few small lesions (less than 3 mm) near cuts; weakly virulent, leaves with chlorosis extending from cuttings; and fully virulent, blackened leaf veins, death, and drying of tissue with V-shape lesions. This rating method was modified from Ignatov *et al*. [87].

For HR tests, *Xcc *strains were cultured as for the virulence assay, adjusted to a density of 10^8 ^colony forming units per ml with distillated water and introduced, by the infiltration method with a needleless syringe [59], into the intercellular spaces of the leaves of non-host plant pepper (*Capsicum annuum *cv. Early Cal Wonder) ECW10R (from Laboratoire de Biologie Moleculaire des relations Plantes Microorganismes INRA-CNRS, Castanet Tolosan, France). After inoculation, the plants were kept at 28°C under continuous illumination of 6,000 lux light intensity. The *ΔhrpG *mutant, a *Xcc *deletion mutant of *hrpG *[88], was used as a negative control.

### Construction of the whole-genome microarray of *Xcc *strain 8004

A high-density PCR-based DNA array was designed by using the genome sequence data of *Xcc *strain 8004 (GenBank accession number CP000050). The genome has 5,148,708 bp and encodes 4,273 predicted CDSs [21]. An in-house high-throughput computer algorithm based on the Linux operating system and Python programming language was employed to design PCR primers for all CDSs. The fundamental rules of our computer algorithm include that all the primer annealing temperatures range from 57.5-68.7°C, and the PCR product sizes fall within 200-1,000 bp, with an optimum of 500 bp. The PCR amplicons should have a minimum sequence similarity with cut-off e-value <1 e^-3 ^and sequence identity <70% when using the BLAST program. There are 87 genes which were designed not to be spotted on the array because of their high sequence similarity to other genes in the genome. The PCR amplifications were performed in a 100 μl reaction volume and PCR success was confirmed by agarose gel electrophoresis. The confirmed PCR products were precipitated with isopropanol and redissolved in DNA Spotting Solution (CapitalBio Corp., Beijing, China). For ORFs that were too small or those genes for which PCR amplification failed, 70-mer sense oligonucleotides were designed; 143 such oligonucleotide probes were synthesized. PCR products and 70-mer oligonucleotides (20 μM) were printed on amino silaned glass slides (CapitalBio Corp.) using a SmartArray™ microarrayer (CapitalBio Corp.). Each CDS was printed in triplicate to facilitate subsequent data analysis. After printing, the slides were baked at 80°C for 1 h and stored dry at room temperature till use. The *Xcc *8004 microarray slides are available to the public from CapitalBio Corp. [89].

Prior to hybridization, the slides were rehydrated over 65°C water for 10 s, and UV cross-linked at 250 mJ/cm^2^. The unimmobilized DNAs were washed off with 0.5% SDS for 15 minutes at room temperature and SDS was removed by dipping the slides in anhydrous ethanol for 30 s. The slides were spin-dried at 1,000 rpm for 2 minutes.

### DNA labeling for aCGH analysis

Genomic DNA was fragmented by *Dpn *II endonuclease digestion, and then purified with the PCR Clean-up NucleoSpin Extract II kits (Macherey-Nagel, Düren, Germany). For each labeling reaction, 2 μg of digested DNA and 4 μg of random nonamer were heated to 95°C for 3 minutes and snap cooled on ice, then 10× buffer, dNTP and Cy5-dCTP or Cy3-dCTP (GE HealthCare Bio-Sciences AB, Björkgatan, Uppsala, Sweden) were added at final concentrations of 120 μM each dATP, dGTP, dTTP, 60 μM dCTP and 40 μM Cy-dye. Klenow enzyme (1 μl; Takara, Dalian, China) was added and the reaction was performed at 37°C for 1 h. The labeled DNA was purified with a PCR Clean-up NucleoSpin Extract II kit, resuspended in elution buffer and checked for its optical density.

### Microarray hybridization, scanning and data analysis

For aCGH, the final products hybridized with microarrays were fluorescence-labeled DNA, so an identical hybridization strategy was employed. Labeled control and test samples were quantitatively adjusted based on the efficiency of Cy-dye incorporation and mixed into 80 μl hybridization solution (3× SSC, 0.2% SDS, 50% formamide). DNA in hybridization solution was denatured at 95°C for 3 minutes prior to loading on the microarray. Hybridization was performed under LifterSlip™ (Erie Scientific Company, Portsmouth, NH, USA), which allows for even dispersal of hybridization solutions between the microarray and coverslip. The hybridization chamber was laid on a Three-phase Tiling Agitator (CapitalBio Corp.) to prompt the microfluidic circulation under the coverslip. The array was hybridized at 42°C overnight and washed with two consecutive washing solutions (0.2% SDS, 2× SSC for 5 minutes at 42°C and 0.2% SSC for 5 minutes at room temperature).

Arrays were scanned with a confocal LuxScan™ scanner (CapitalBio Corp.) and the data of obtained images were extracted with SpotData software (CapitalBio Corp). In order that the aCGH results were also represented with the fluorescence intensity ratio, a spatial and intensity-dependent normalization based on a LOWESS program was employed, which is prevalent in microarray expression profiling [31]. Since each gene was represented in triplicate on each slide and the experiments were performed in duplicate by dye swap, producing six data points, the average ratio (always sample/reference strain 8004) of each gene was input into hierarchical clustering with an average linkage algorithm for aCGH analysis.

All the aCGH data can be accessed at the National Center for Biotechnology Information Gene Expression Omnibus (GEO) database [90] with accession number GSE5087.

Putative AHD CDSs identified by aCGH were examined by PCR using the primers designed within CDSs in strain 8004. The oligonucleotide primers and the PCR results are shown in Additional data file 3.

### Bioinformatic analysis

Whole genome comparison of the CDS set of strain 8004 with that of each sequenced xanthomonad strain was carried out using the BLASTN program [91]. Shared genes were defined using an e-value cutoff of e^-20^. The CDS sets were obtained from GenBank with the following accession numbers (in parentheses): *Xcc *8004 (CP000050), *Xcc *ATCC33913 (AE008922), *X. axonopodis *pv. *citri *(*Xac*) 306 and plasmids pXAC33 and pXAC64 (AE008923, AE008924 and AE008925, respectively), *X. oryzae *pv. *oryzae *(*Xoo*) KACC10331 (AE013598), *Xoo *MAFF311018 (NC_007705), *X. campestris *pv. *vesicatoria *(*Xcv*) strain 85-10 and its four plasmids pXCV2, pXCV19, pXCV38, and pXCV183 (AM039948, AM039949, AM039950, AM039951, and AM039952, respectively).

The phylogenetic relationships of all the *Xcc *strains tested and other xanthomonad strains used as references were constructed by the maximal parsimony method based on pairwise comparisons of partial 16S-23S rDNA ITSs, which were obtained from direct ITS rDNA sequencing of Chinese strains and from GenBank with the accession number of each xanthomonad strain: *X. axonopodis *pv. *aurantifolii *(*Xaa*) strain X84 (AF442739.1), *Xcc *strain XCC15 (AF123092.2), *X. axonopodis *pv. *dieffenbachiae *(*Xad*) ATCC23379 (AY576642.1), *Xad *X195 (AY576648.1), *X. arboricola *pv. *pruni *(*Xap*) (AJ936965.1), *X. gardneri *(*Xg*) strain CNPH496 (AY288083.1), *X. vesicatoria *(*Xv*) strain CNPH345 (AY288080.1), *Xv *XV1111 (AF123088.2), and other strains, such as *Xcc *8004, *Xcc *ATCC33913, *Xac *306, *Xcv *85-10, *Xoo *KACC10331, and *Xoo *MAFF311018 with the same accession number as that for each genome.

The genomic dissimilarity δ* values (the average dinucleotide relative abundance difference) between the putative variable genomic region in *Xcc *(XVR) and the genome sequence of *Xcc *strain 8004 were determined by the δρ-WEB program [55,92] and are listed in Table 5. A BLASTN search in GenBank was carried out for each XVR in order to identify the origin of potential horizontal gene transfer if the homology was high enough.

### Correlation analysis

To identify the genetic determinants for host specificity of *Xcc*, a correlation analysis was performed using the CORREL tool in Excel (Microsoft Office 2000). Prior to statistical operation, the aCGH result of each gene in any *Xcc *strain was transformed from the ratio value to the numerical code: 0 = absent or highly divergent; 1 = present. The pathogenicity test results were transformed from a qualitative description to a numerical code: 0 = non-pathogenic; 1 = pathogenic. The HR results were transformed from a qualitative description to a numerical code: 0 = no HR; 1 = HR. For one round of statistical operation, a direct correlation analysis between virulence scales of the 20 *Xcc *strains (including 18 Chinese stains, strain ATCC33913 and strain 8004) on one given plant cultivar and the distribution pattern of each gene in 20 *Xcc *strains was carried out using the program CORREL. Twelve operations were performed and the CC values of each operation were listed in one column, parallel to the gene list of strain 8004 (Additional data files 8 and 9).

In each correlation analysis (for each plant assay), the *Xcc *genes with the maximal R absolute values were selected as the candidates responsible for host specificity of *Xcc *strain 8004 on a particular plant. Due to the possibility of more than one gene having the same distribution pattern among 20 *Xcc *strains, more than one candidate gene for each genetic determinant was able to be selected (Table 10).

### Construction of the T4SS-deletion mutant of *Xcc*

The *virB/D4 *T4SS deletion mutant was generated by the marker exchange method. The upstream and downstream fragments flanking the *virB/D4 *cluster were amplified with the primer sets DT4-LF/DT4-LR (Additional data file 1) (the coordinate position of the amplified fragment in *Xcc *8004 chromosome is from 1956072 to 1957097, and DT4-RF/DT4-RR (the coordinate position of the amplified fragment in *Xcc *8004 chromosome is from 1965913 to 1966832, respectively). Simultaneously, the gentamicin resistant fragment was amplified with the primer sets Gm-F/Gm-R (Additional data file 1). The obtained fragments were cloned into the *Eco*RI-*Kpn*I-*Bam*HI-*Xba*I sites of the suicide vector pK18*mobsacB *[70] one by one, yielding the recombinant plasmid pKDT4. The plasmid pKDT4 was transferred into *Xcc *wild-type strain 8004 by triparent conjugation and kanamycin resistant transconjugant colonies were screened. Bacterial cells cultured in NYG broth without antibiotics overnight from a single transconjugant colony chosen randomly were diluted gradiently and plated on the NYG agar plats with 5% sucrose and appropriate gentamicin. The gentamicin resistant and kanamycin sensitive colonies were screened, generating the deletion mutant of *virB*/*D4 *T4SS, named 8004ΔT4 (Figure 5). The deletion mutant 8004ΔT4 was further confirmed by PCR with the primer sets DT4-LF/DT4-RR (Additional data file 1) and the primer sets of each ORF of *virB*/*D4 *T4SS (Additional data file 7). The virulence of the mutant was tested on host plants by the leaf-clipping inoculation method [85].

### Functional analysis of genetic determinants for host specificity

The candidate *avr *genes (*XC2602*, *XC2004 *and *XC2081*) of *Xcc *8004 were disrupted by using the plasmid pK18*mob*, a conjugative suicide plasmid in *Xcc *[70]. The internal fragment of each target gene was amplified by PCR using chromosomal DNA of *Xcc *strain 8004 as template and the primers designed according to certain CDSs (Additional data file 1), and cloned into the plasmid pK18*mob *to generate a recombinant plasmid. The identity of the cloned fragment was confirmed by sequencing. Each recombinant plasmid was transformed into *Escherichia coli *JM109 (Additional data file 1) and then introduced into the wild-type strain 8004 by triparental conjugation using the helper plasmid pRK2073 (Additional data file 1). Transconjugants were selected on the NYG plates containing rifampicin and kanamycin. Mutants were screened for disruption of the target gene by PCR using primer PMOB-SP (Additional data file 1), a specific primer from pK18*mob*, and a specific primer of the upstream gene of each target gene (Additional data file 1). The obtained mutants of *XC2004*, *XC2602 *and *XC2081 *were named NK2004, NK2602 and NK2081, respectively.

The complementation of the mutation of each target gene was carried out by introduction of the broad host range cosmid pLAFR3 carrying the intact target gene into the corresponding mutant strain. The intact target gene was amplified by PCR using chromosomal DNA of *Xcc *8004 as template and the specific primer sets (Additional data file 1), and cloned into the plasmid pLAFR3 under the control of the P_lac _promoter. The identity of the cloned target gene was confirmed by sequencing. The confirmed recombinant plasmid was transformed into *E. coli *JM109 and then introduced into the corresponding mutant strain by triparental conjugation. The transconjugants were screened on NYG plates with rifampicin, kanamycin and tetracycline. The created complementary strains for the mutants NK2602, NK2004 and NK2081 were named CNK2602, CNK2004 and CNK2081, respectively.

For verification of the *avr *function of putative *avrXccE1*, the plasmid containing *XC2602 *was transferred into the Chinese strains CN01, CN05, CN10 and CN11, which contain no homologs of *XC2602*. A 1,605 bp fragment that includes the region from 514 bp upstream of the star codon to 29 bp downstream of the stop codon of *XC2602 *was amplified with the primer set XC2602CM-F/XC2602CM-R (Additional data file 1) using the total DNA of *Xcc *8004 as template. After confirmation by sequencing, the fragment was cloned into the promoterless cosmid pLAFR6 to generate the recombinant plasmid named pC2602. The recombinant plasmid pC2602 was transferred into the strains CN01, CN05, CN10 and CN11 by triparental conjugation. The transconjugants carrying pC2602 were screened on NYG plates with rifampicin and tetracycline, and named CN01/pC2602, CN05/pC2602, CN10/pC2602 and CN11/pC2602, respectively. The virulence of the obtained strains CN01/pC2602, CN05/pC2602, CN10/pC2602 and CN11/pC2602 on Chinese cabbage cv. Zhongbai-83 was tested by the leaf-clipping method described above.

## Abbreviations

aCGH, array-based comparative genome hybridization; AHD, absent/highly divergent; CC, correlation coefficient; CDS, coding sequences; cv., cultivar; HR, hypersensitive response; ITS, intergenic spacer; LPS, lipopolysaccharide; ORF, open reading frame; T4SS, type IV secretion system; *Xcc*, *Xanthomonas campestris *pathovar *campestris*; XVR, *Xanthomonas *variable genomic region.

## Authors' contributions

JLT and YQH were responsible for strategic planning and managing the overall project. LZ, BLJ, JC, and XXL constructed the microarray and performed the aCGH analyses. RQX, SSZ, GTL and JQ performed the isolation and characterization of the Chinese *Xcc *strains. BLJ, RQX, ZCZ, MLW and JXF constructed the mutants of the putative *avr *genes and the T4SS deletion mutant. DJT, JRC, XZ and JL performed plant assays. LZ, WJ and YQH performed the bioinformatic analysis. JLT, YQH and BC performed CC and other data analyses. JLT, YQH and LZ wrote the paper. All authors have read and approved the final manuscript.

## Additional data files

The following additional data are available with the online version of this paper. Additional data file 1 contains Tables S1 and S2, which summarize the bacterial strains and plasmids and the primers used in this study, respectively. Additional data file 2 is a figure showing a maximal parsimony dendrogram depicting phylogenetic relationships of partial 16S-23S rDNA ITS sequences of all of the Chinese *Xcc *strains examined and other *Xanthomonas *spp. Additional data file 3 is a figure illustrating the confirmation of some present or AHD genes defined by aCGH. Additional data file 4 is a table presenting detailed data on the aCGH results. Additional data file 5 is a table showing the re-annotation of genes from *XC2070 *to *XC2086 *in the genome of *Xcc *strain 8004. Additional data file 6 is a table listing the 305 proven/presumed pathogenicity genes among *Xcc *strains revealed by aCGH analyses. Additional data file 7 is a figure showing the deletion and confirmation of the T4SS locus in *Xcc *8004. Additional data file 8 contains the numerical codes transferred from the results of aCGH analyses and plant tests. Additional data file 9 is a table presenting the coefficient values of correlation between plant test results and the gene distribution patterns of *Xcc *strains.

## Supplementary Material

Additional data file 1 Bacterial strains and plasmids and the primers used in this study. Click here for file

Additional data file 2 Phylogenetic relationships of partial 16S-23S rDNA ITS sequences of all of the Chinese *Xcc *strains examined and other *Xanthomonas *spp. Click here for file

Additional data file 3 Confirmation of some present or AHD genes defined by aCGH. Click here for file

Additional data file 4 Array-based comparative genome hybridization results. Click here for file

Additional data file 5 Re-annotation of genes from *XC2070 *to *XC2086 *in the genome of *Xcc *strain 8004. Click here for file

Additional data file 6 The 305 proven/presumed pathogenicity genes among *Xcc *strains revealed by aCGH analyses. Click here for file

Additional data file 7 Deletion and confirmation of the T4SS locus in *Xcc *8004. Click here for file

Additional data file 8 Numerical codes transferred from the results of aCGH analyses and plant tests. Click here for file

Additional data file 9 Coefficient values of correlation between plant test results and the gene distribution patterns of *Xcc *strains. Click here for file
